# Morphological and Phylogenetic Evidence Reveal Nine New Species of *Russula* (Russulaceae, Russulales) from Shanxi Province, North China

**DOI:** 10.3390/jof12010078

**Published:** 2026-01-22

**Authors:** Hao-Yu Fu, Jia-He Li, Hui-Min Ji, Ning Mao, Ting Li, Li Fan

**Affiliations:** 1 College of Life Sciences, Capital Normal University, Xisanhuanbeilu 105, Haidian, Beijing 100048, China; 2220801018@cnu.edu.cn (H.-Y.F.); 2240802077@cnu.edu.cn (J.-H.L.); thekingj99@163.com (H.-M.J.); 2 The Institute of School of Life Science, Shanxi University, Taiyuan 030006, China; maoning@sxu.edu.cn; 3 Department of Life Sciences, National Natural History Museum of China, Tianqiaonandajie 126, Beijing 100050, China; m_zhou_m@163.com

**Keywords:** basidiomycetes, morphology, multi-locus analysis, phylogeny, taxonomy

## Abstract

Shanxi Province, located in northern China, characterized by a warm-temperate monsoon climate, complex mountainous topography, and vegetation dominated by trees of Fagaceae and Pinaceae, provides diverse habitats for *Russula* diversity. Recent investigations on macrofungi in this region revealed nine new *Russula* species based on integrated morphological and multi-locus phylogenetic analyses (ITS, nrLSU, rpb2, tef1), which are described and illustrated in this paper. These new taxa are classified into three subgenera of *Russula*: one species of subgen. *Brevipes*, four of subgen. *Heterophyllidia*, four of subgen. *Russula*. This work enhances the understanding of *Russula* resources in China’s temperate zone.

## 1. Introduction

The diversity of macrofungi plays a fundamental role in forest ecosystems, yet a significant portion of this diversity remains undocumented. *Russula* Pers. is one of the most species rich fungal genera, and key components in ectomycorrhizal (ECM) forests worldwide, estimated at least to contain 2000–3000 species [1,2,3,4]. Many *Russula* species are harvested across the world as delicious edible mushrooms; meanwhile, some species are toxic including a few of lethal like *R. subnigricans* [5,6,7,8,9,10,11,12,13,14]. The monophyly of *Russula* is settled by Buyck et al. (2008) [15] based on multilocus DNA data, and the latest classification system of *Russula* contains nine subgenera, *viz*. *Russula* subg. *Archaeae* Buyck and V. Hofst., *R*. subg. *Brevipedum* Buyck and V. Hofst., *R.* subg. *Compactae* (Fr.) Bon, *R.* subg. *Crassotunicatae* Buyck and V. Hofst., *R.* subg. *Cremeoochraceae* Buyck and X.H. Wang, *R.* subg. *Glutinosae* Buyck and X.H. Wang, *R.* subg. *Heterophyllidiae* Romagn., *R.* subg. *Malodorae* Buyck and V. Hofst. and *R.* subg. *Russula* Pers. [2,5,16,17,18,19].

Shanxi Province is located in the northern area of China and is one of the Chinese provinces with warm-temperate monsoon climate. The annual average temperature is 5–10 °C. The annual average rainfall is 300–600 mm. The terrain of Shanxi Province is mountainous and hilly, with an average altitude of well over 1500 m above sea level. The highest peak (located in Wutaishan Mountains, northern Shanxi Province) stands at an elevation of 3058 m. The natural vegetation consisted of deciduous broad-leaved forest, coniferous and broad-leaved mixed forest, coniferous forest, and grassland, in which some plants of ectomycorrhizal-association with *Russula* species dominate, such as species of *Betula*, *Quercus*, *Larix*, *Picea*, *Pinus*, *Populus.*

Recent years, in the ongoing investigations on macrofungi of Shanxi Province [20,21,22,23,24,25,26,27,28,29,30,31,32], a lot of *Russula* specimens were collected from this area. Among them, nine new species of *Russula* were recognized based on morphological and phylogenetic analyses, which are, respectively, classified into three subgenera of *Russula*, *viz.* subgen. *Brevipes*, subgen. *Heterophyllidia*, and subgen. *Russula*. The aim of this paper is to describe and illustrate these new species.

## 2. Materials and Methods

### 2.1. Morphological Studies

Fresh specimens were collected between 2019 and 2024 from Shanxi Province, North China. Fresh basidiocarps were obtained and photographed in the field, then dried in a fruit drier at 45 °C, and deposited in BJTC (Herbarium, Biology Department, Capital Normal University). Standardized color values matching the color of the description were taken from ColorHexa (http://www.colorhexa.com/). Macroscopic features, such as color, size, shape, and stipe, were documented based on fresh specimens. Microscopic features were observed using dried material, with free-hand sections of basidiocarps mounted in 5% KOH, basidiospores were observed and measured in Melzer’s reagent and cystidia were observed and measured in sulphovanillin. For the measurements of the basidiospores, the abbreviation [n/m/p] means ‘n’ basidiospores measured from ‘m’ basidiomes of ‘p’ collections; ‘Q’ is used to mean “length/width ratio” of a spore in side-view. ‘Qm’ refers to the average Q of all basidiospores, including sample standard deviation. The dimensions of basidia and cystidia were based on 30 of them each. The measurement of basidia excluded the length of sterigmata. Microscopic features were examined using an Olympus BX51 light microscope (Olympus Corporation, Tokyo, Japan) at magnifications ranging from 400 to 1000× [5]. For scanning electron microscopy (SEM), spores were scraped from the dried lamellae, placed onto double-sided tape that was mounted directly on the SEM stub, coated with a platinum–palladium film 8 nm thick using an ion-sputter coater (HITACHI E-1010) (Hitachi High-Technologies Corporation, Tokyo, Japan), and examined with a HITACHI S-4800 SEM (Hitachi High-Technologies Corporation, Tokyo, Japan).

### 2.2. DNA Extraction, PCR Amplification and DNA Sequencing

From dried basidiocarps, 10–30 mg of dry tissue was taken and subjected to cryogenic grinding. Total genomic DNA was then extracted using the modified CTAB protocol [33]. The internal transcribed spacer region of nuclear ribosomal DNA (ITS) was amplified using primers ITS1F/ITS4 [33,34]. The 28S large subunit of nrDNA region (nrLSU) was amplified using primers LR0R/LR5 [35]. RNA polymerase II largest subunit (rpb2), and translation elongation factor 1-alpha (tef1) were following Buyck et al. (2018, 2020) [2,17]. PCRs were performed in 50 μL reactions containing 2 μL DNA template, 1 μL primer (10 μM) each, 25 μL of 2 × Master Mix (Tiangen Biotech (Beijing) Co. (Beijing, China)), 17 μL ddH_2_O, 4 μL dNTP. The thermal cycler conditions for ITS and nrLSU markers were initial denaturation at 94 °C for 3 min; 35 cycles of denaturation at 94 °C for 30 s, annealing at 52 °C (ITS) or 58 °C (nrLSU) for 45 s, and extension at 72 °C for 1 min; followed by a final extension at 72 °C for 10 min [36]. The thermal cycler conditions for rpb2 and tef1 were followed the protocol described by Buyck et al. (2024) [5]. Amplified PCR products were sent to Beijing Zhongkexilin Biotechnology Co. Ltd. (Beijing, China) for purifying, sequencing. The generated raw reads of the DNA sequences were used to obtain consensus sequences using SeqMan v.7.1.0 in the DNASTAR Lasergene Core Suite software v7.1 (DNASTAR Inc., Madison, WI, USA).

### 2.3. Phylogenetic Analysis

The sequences used for phylogenetic analysis are in Supplements 1–5, with newly generated sequences in this study highlighted in bold. Apart from the newly generated sequences, other sequences used in this study were retrieved from GenBank database by BLASTn v2.17.0 search with a significant similarity, or selected from those used by Buyck et al. (2018, 2020), Zhou et al. (2020), Song et al. (2022), Han et al. (2023), Xie et al. (2023) and Zhou et al. (2023) [2,17,37,38,39,40,41].

Six datasets were used to analyze phylogenetic position of the new species of three subgenera of *Russula* from Shanxi, China, respectively, marked as Dataset I–VI. Dataset I (ITS) and Dataset II (ITS-nrLSU-rpb2) were assembled for subgenus *Brevipes*; Dataset III (ITS) and Dataset IV (ITS-nrLSU) for subgenus *Heterophyllidia*; Dataset V (ITS) and Dataset VI (nrLSU-rpb2-tef1) for subgenus *Russula*. Dataset I, III, and V were designed to encompass a broader range of species, including those in the database for which only the ITS locus is available. Dataset II, IV, and VI were constructed to determine their precise phylogenetic position. Based on previous studies on the phylogeny of Russulaceae [2,17], the selection of outgroups for the ITS datasets (Dataset I, III, and V) followed Buyck et al. (2018) [2]; the outgroup for the other three combined datasets (Dataset II, IV, and VI) adhered to Zhou et al. (2020, 2023), Song et al. (2022), Xie et al. (2023), Han et al. (2023) [37,38,39,40,41]. Sequences were aligned in MAFFT v.7.110 [42] under default parameters, and manually adjusted to allow maximum sequence similarity in Se-Al version.2.03a [43]. Phylogenetic analyses were conducted using maximum likelihood (ML) and Bayesian Inference (BI).

ML analysis was performed using RAxML 8.0.14 [44] under the GTRGAMMA model with 1000 bootstrap replicates for all six datasets. BI analysis was conducted using MrBayes v3.1.2 [45] with a partitioned mixed model (each marker treated as separate partitions). Optimal substitution models for each partition were determined using MrModeltest v2.3 [46] under the Akaike information criterion (AIC). For the concatenated analysis (Dataset II, IV, VI), each locus is considered a partition and assigned its own best-fitting substitution model, that is GTR + I + G for ITS and GTR + I + G for nrLSU in Dataset II (subg. *Brevipes*); GTR + I + G for ITS, GTR + I + G for nrLSU and K80 + G for rpb2 in Dataset IV (subg. *Heterophyllidia*); SYM + I + G for rpb2, HKY + I + G for tef1-α and GTR + I + G for nrLSU in Dataset VI (subg. *Russula*). An independent run with four Markov chains Monte Carlo (MCMC) was conducted for 1,690,000 generations (Dataset II), 3,015,000 generations (Dataset IV), 2,655,000 generations (Dataset VI) under the default settings. For single gene analysis (Dataset I, III, and V), the best substitution model for each dataset, respectively, was GTR + I + G (Dataset I), HKY + I + G (Dataset III), and GTR + I + G (Dataset V). The MCMC analysis was run for 955,000 generations (Dataset I), 1,585,000 generations (Dataset III), 2,950,000 generations (Dataset V). Average standard deviations of split frequency values were far less than 0.01 at the end of the generations. Trees were sampled every 100 generations after burn-in (well after convergence), and a 50% majority-rule consensus tree was constructed. Trees were visualized with TreeView version 1.6.6 [47]. Clades with bootstrap support (BS) ≥70% and Bayesian posterior probability (PP) ≥0.97 were considered significant [48,49].

## 3. Results

### 3.1. Molecular Phylogenetics

A total of 69 new sequences were generated for this study (31 ITS, 19 nrLSU, 10 rpb2 and 9 tef1) and deposited in GenBank (see Supplement 1).

**Figure 1:**
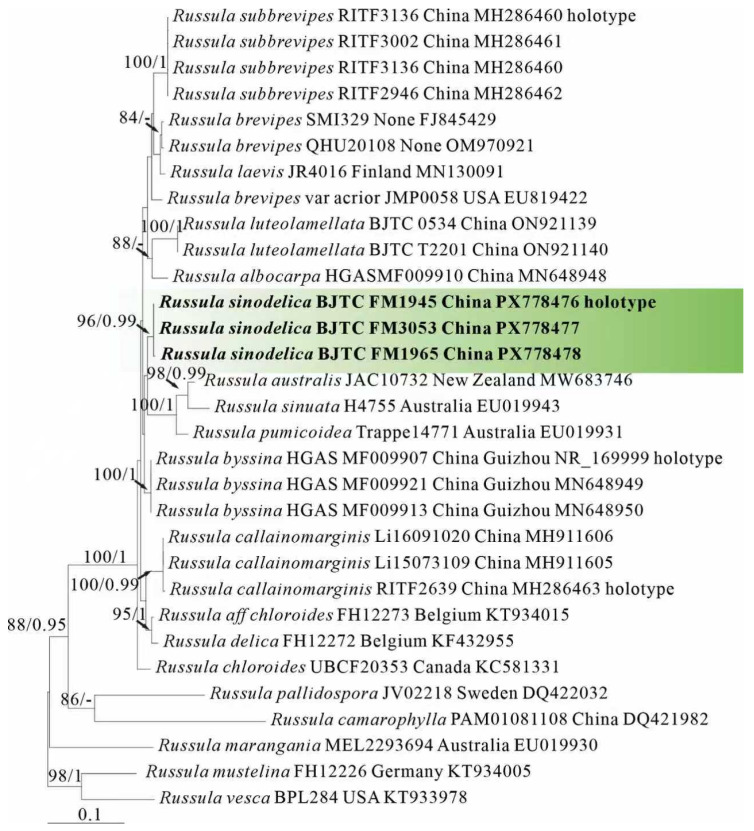
Phylogeny from the maximum likelihood analysis based on ITS sequences (Dataset I) from *Russula* subgen. *Brevipes. Russula mustelina* and *R. vesca* served as outgroups. Values of likelihood bootstrap support values (≥70%, left) and Bayesian significant posterior probabilities (≥0.97, right) are indicated above the nodes. New species and new sequences are in bold. New species are in bold and shaded in green. The holotype specimens of the species involved in this study have been labeled.

**Figure 2:**
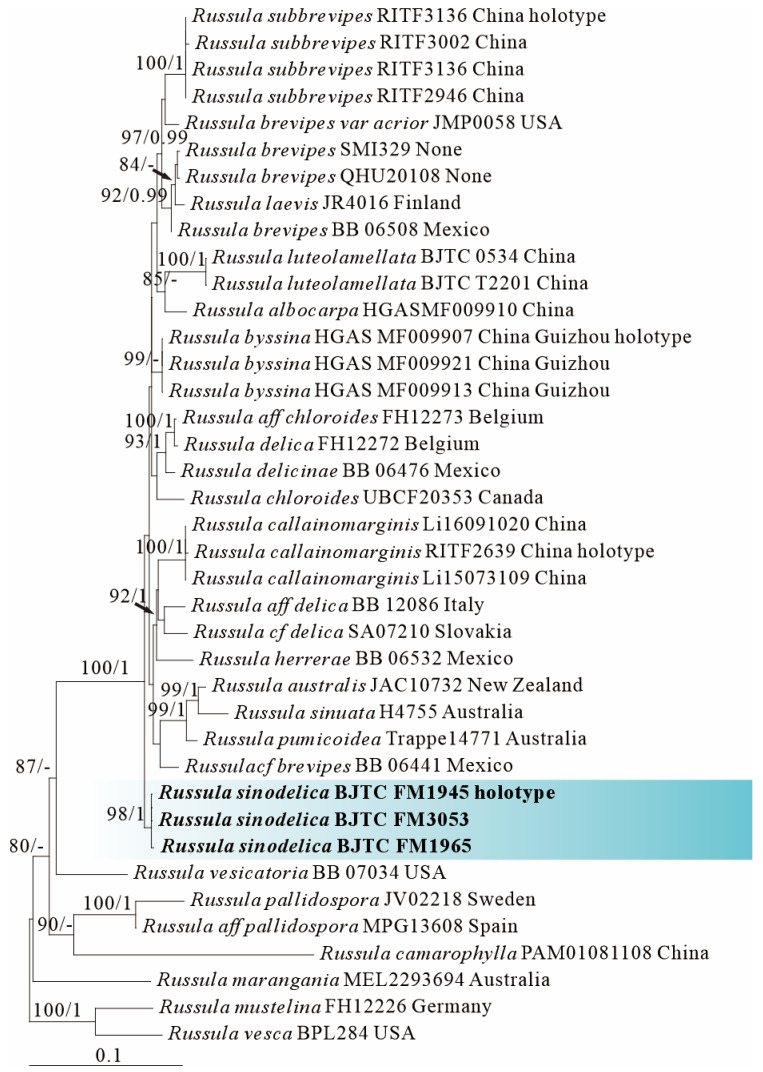
Phylogeny from the maximum likelihood analysis based on ITS, nrLSU and rpb2 sequences (Dataset II) from *Russula* subgen. *Brevipes. Russula mustelina* and *R. vesca* served as outgroups. Values of likelihood bootstrap support values (≥70%, left) and Bayesian significant posterior probabilities (≥0.97, right) are indicated above the nodes. New species and new sequences are in bold. New species are shaded in a blue color. The holotype specimens of the species involved in this study have been labeled.

**Figure 3:**
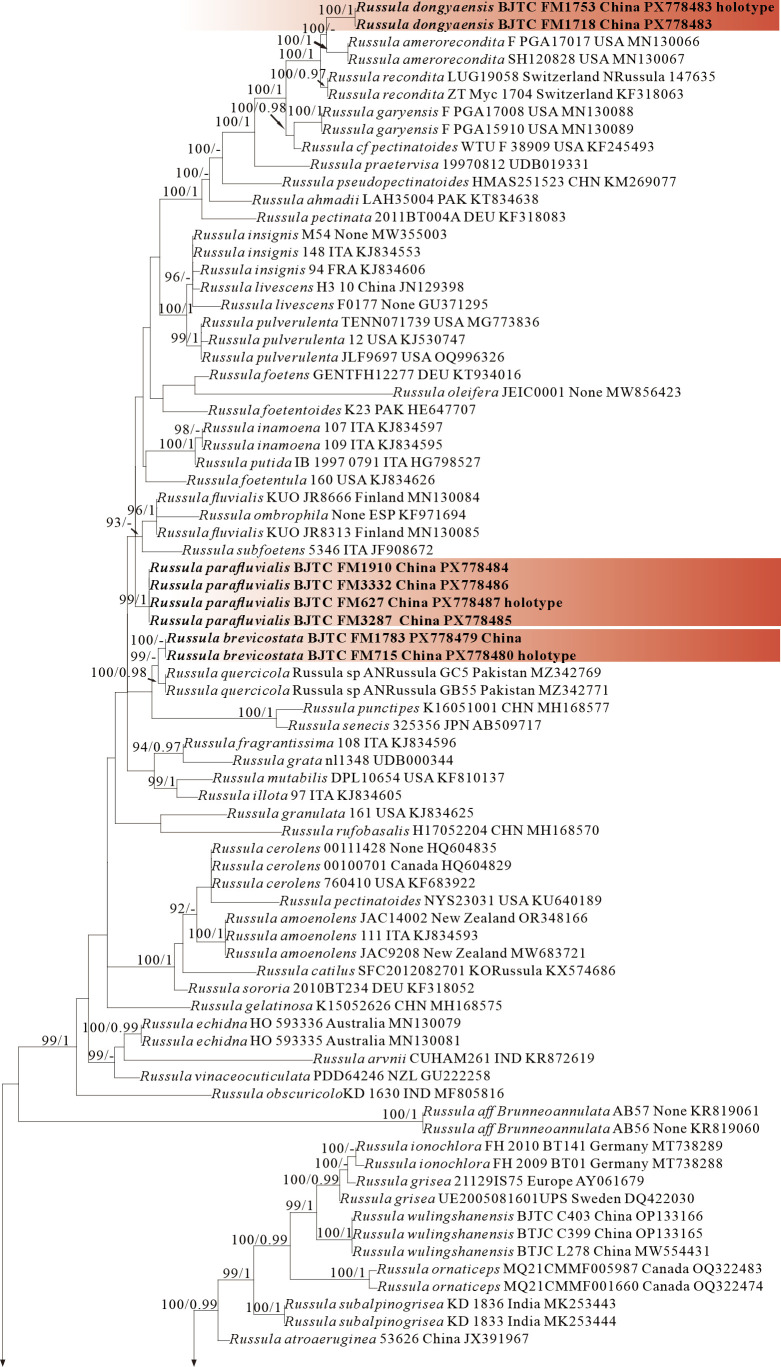

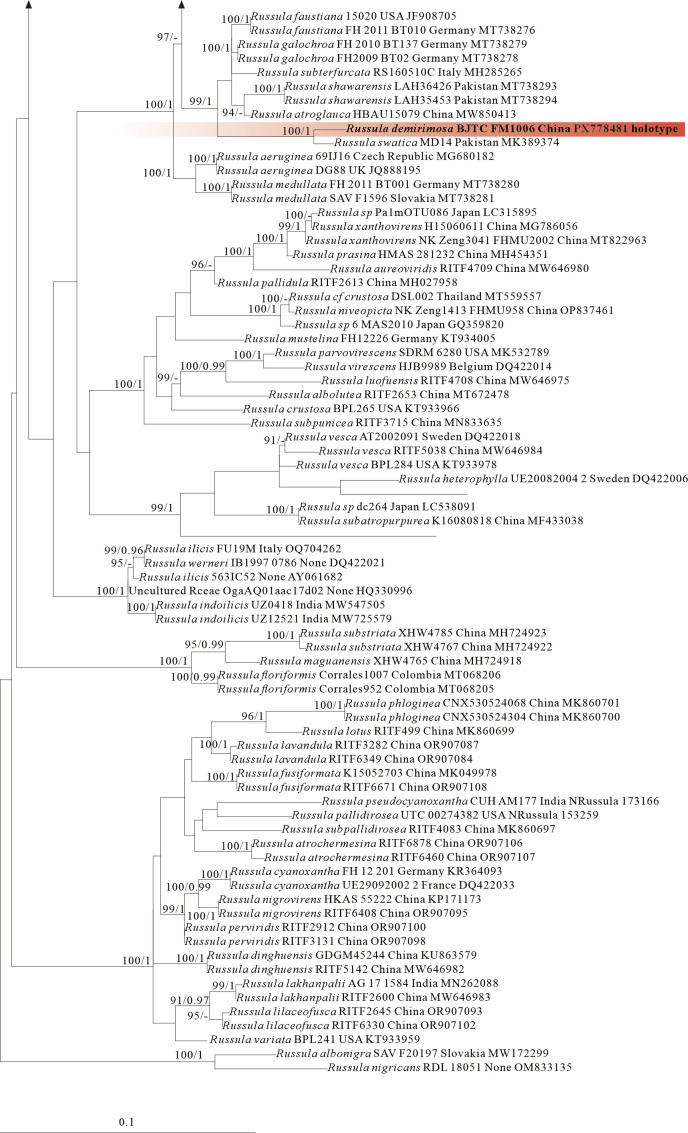
Phylogeny from the maximum likelihood analysis based on ITS sequences (Dataset III) from *Russula* subgen. *Heterophyllidia. Russula maguanensis* and *R. substriata* as outgroups. Values of likelihood bootstrap support values (≥70%, left) and Bayesian significant posterior probabilities (≥0.97, right) are indicated above the nodes. New species and new sequences are in bold. New species are shaded in a red color.

**Figure 4:**
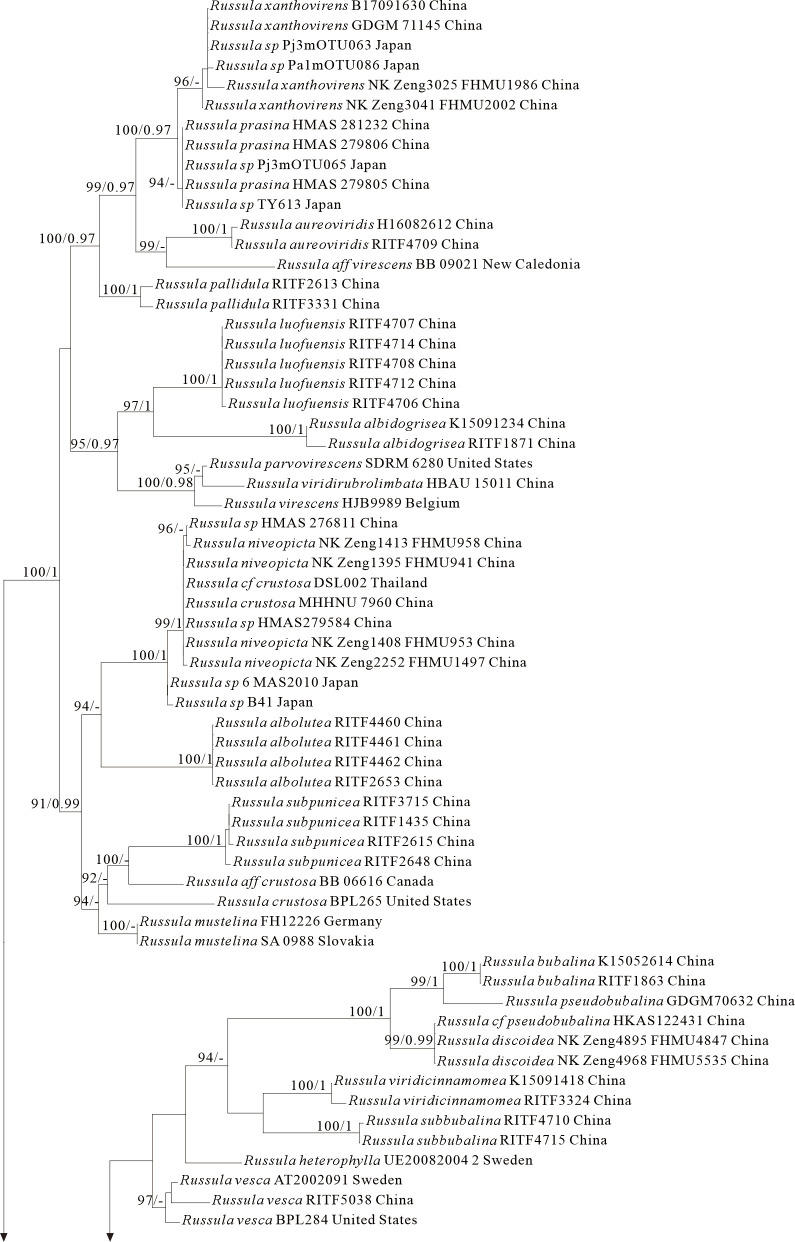

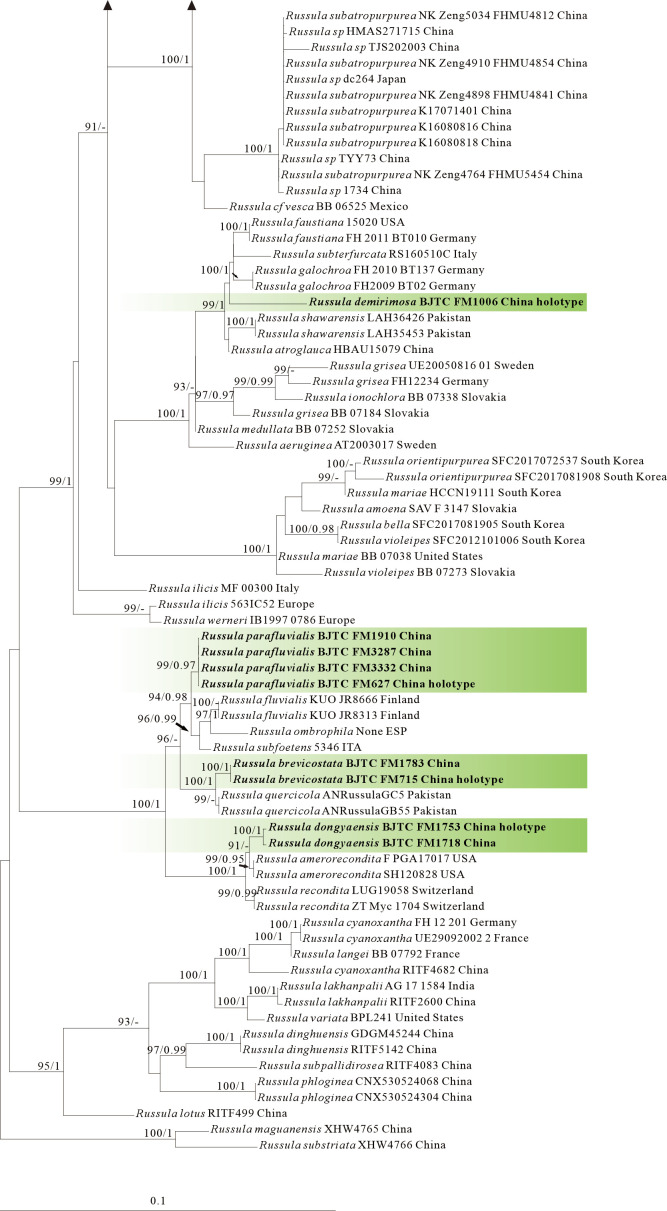
Phylogeny from the maximum likelihood analysis based on ITS and nrLSU sequences (Dataset IV) from *Russula* subgen. *Heterophyllidia. Russula maguanensis* and *R. substriata* served as outgroups. Values of likelihood bootstrap support values (≥70%, left) and Bayesian significant posterior probabilities (≥0.97, right) are indicated above the nodes. New species and new sequences are in bold. New species are shaded in a green color.

**Figure 5:**
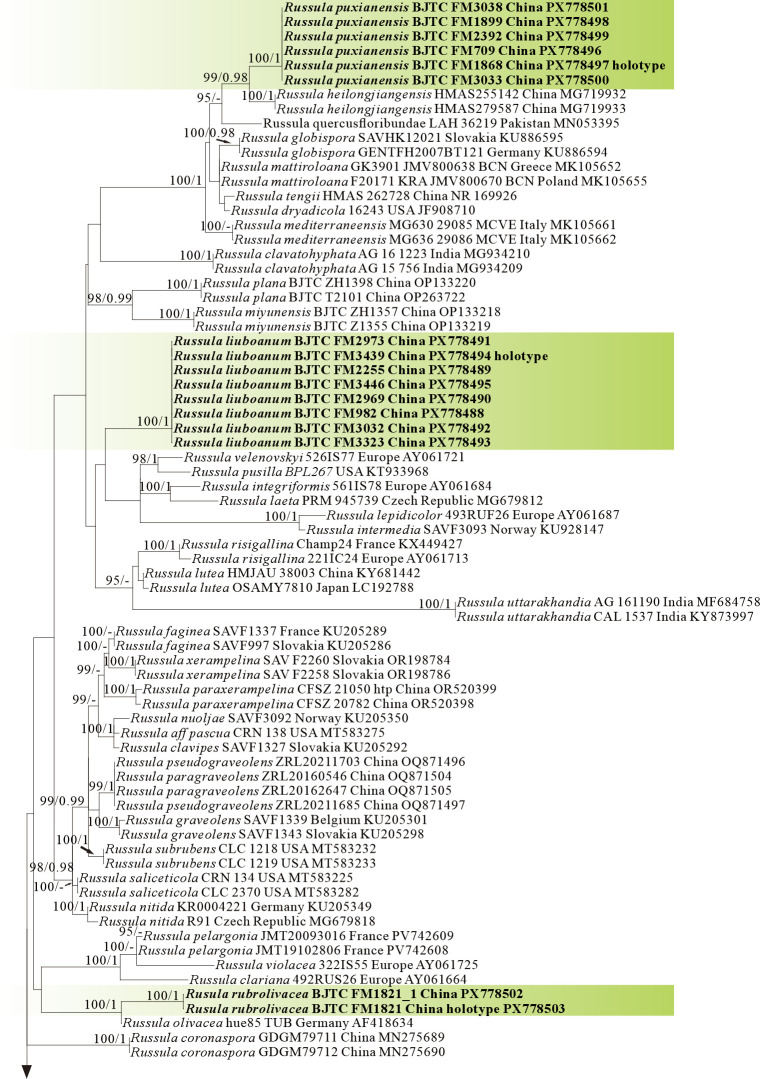

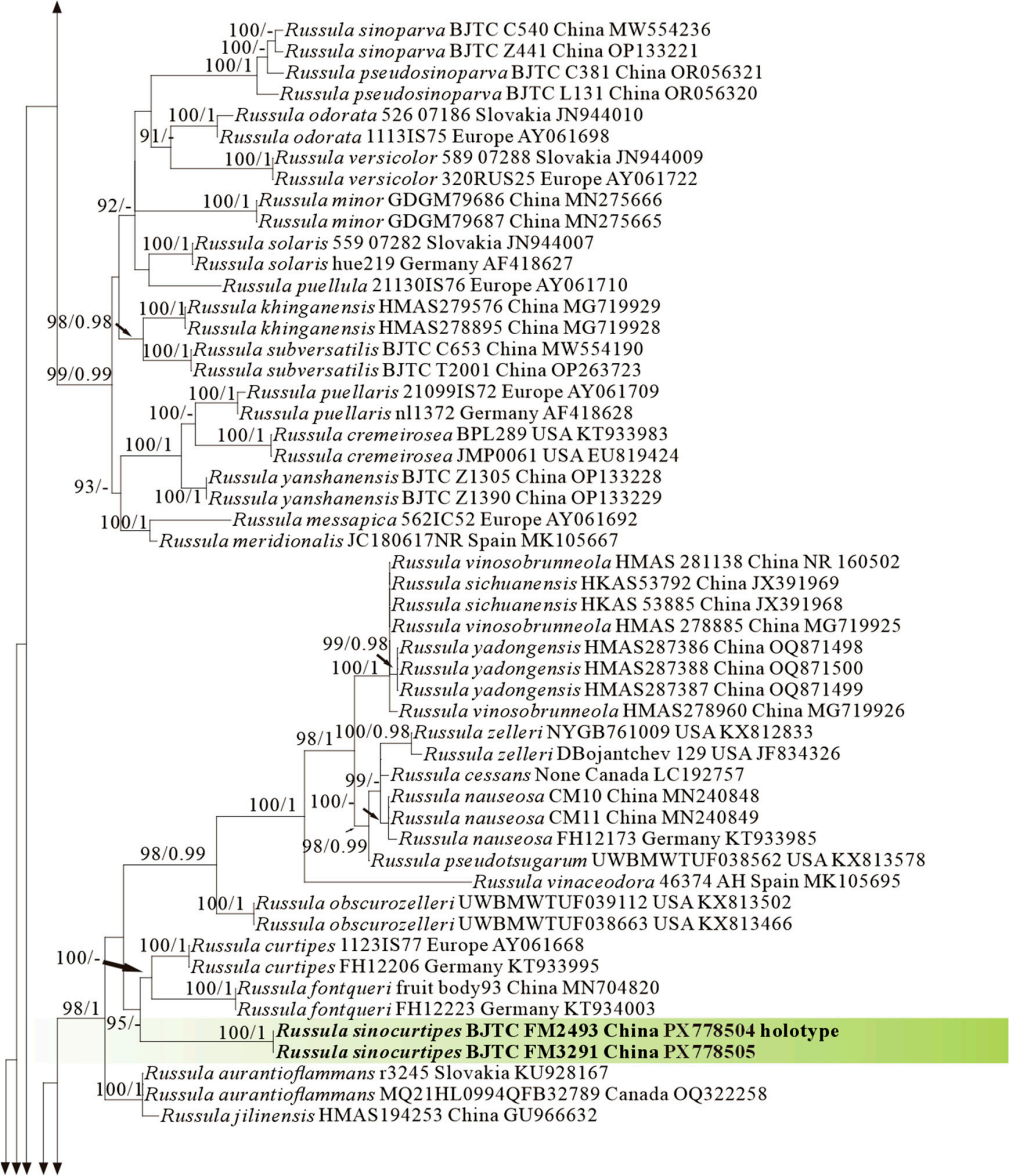

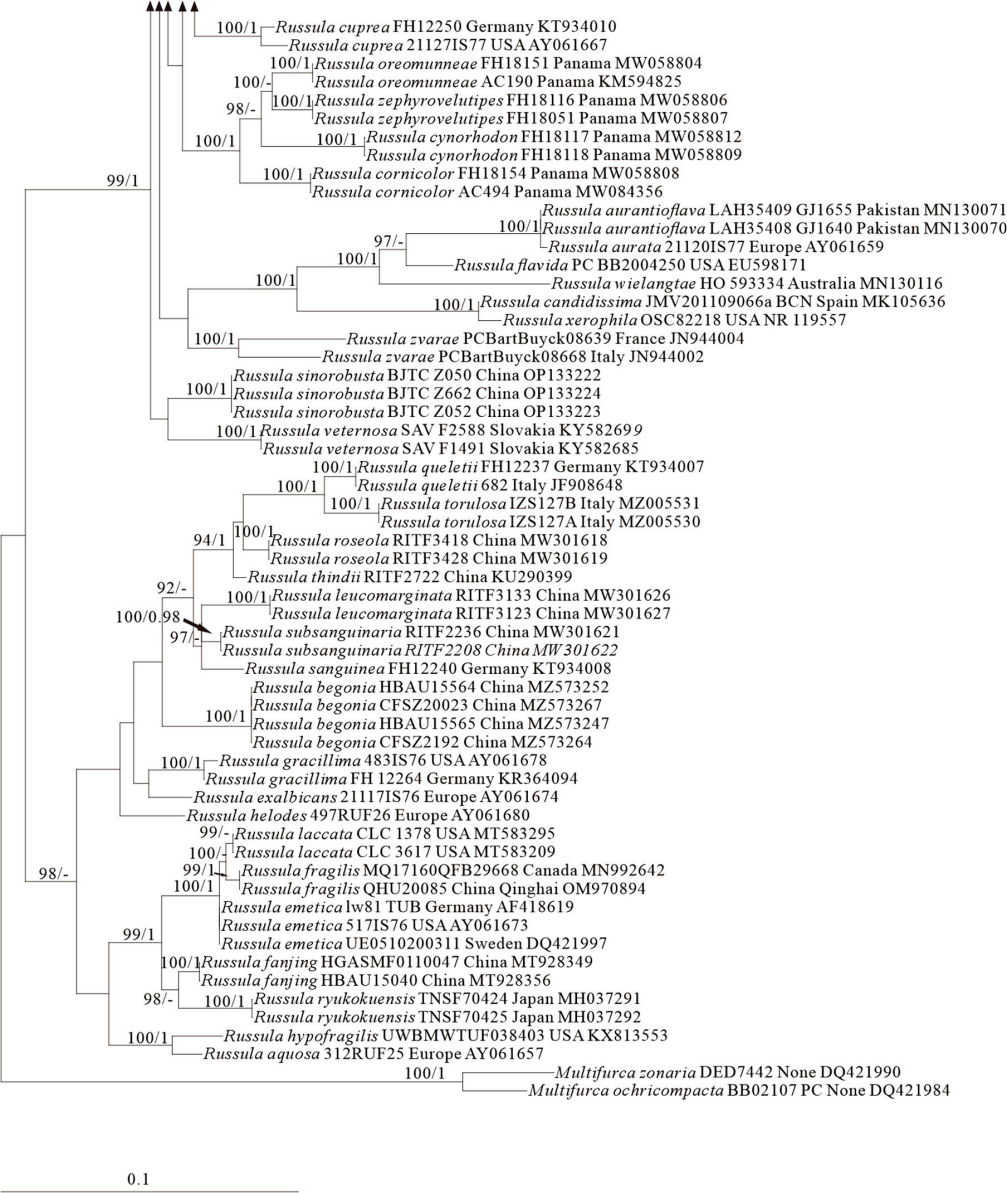
Phylogeny from the maximum likelihood analysis based on ITS sequences (Dataset V) from *Russula* subgen. *Russula*. *Multifurca zonaria* and *M. ochricompacta* served as outgroups. Values of likelihood bootstrap support values (≥70%, left) and Bayesian significant posterior probabilities (≥0.97, right) are indicated above the nodes. New species and new sequences are in bold. New species are shaded in a green color. The holotype specimens of the species involved in this study have been labeled.

**Figure 6:**
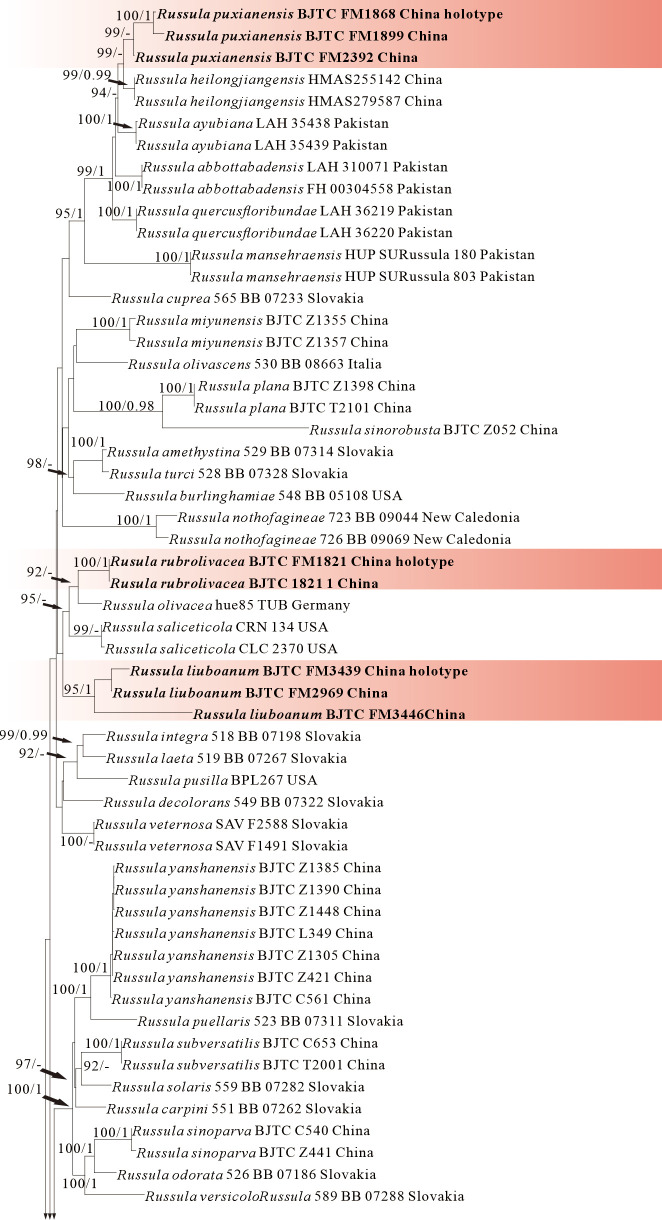

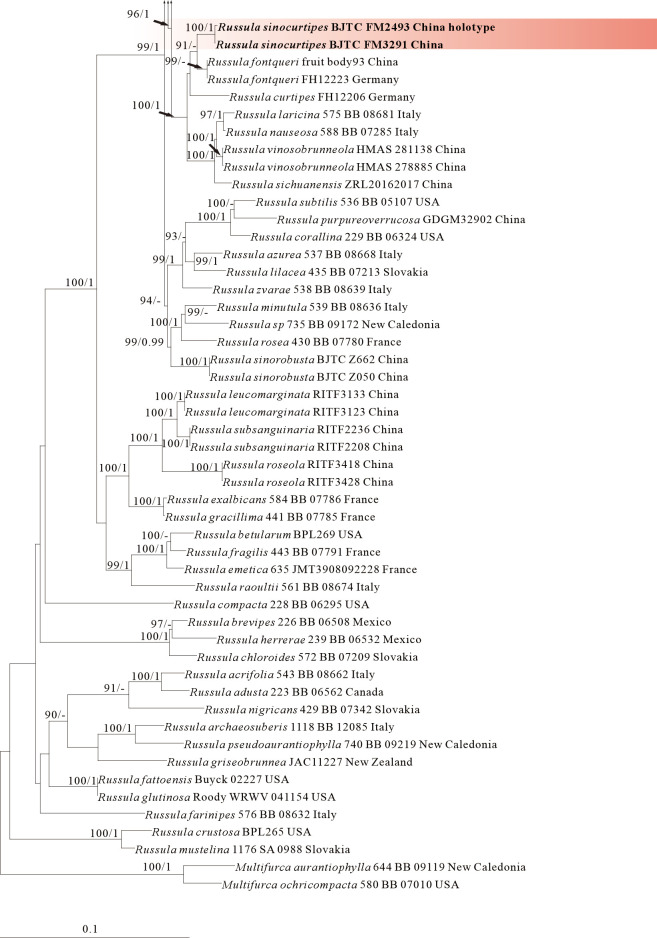
Phylogeny from the maximum likelihood analysis based on nrLSU rpb2 and tef1 sequences (Dataset VI) from *Russula* subgen. *Russula. Multifurca zonaria* and *M. ochricompacta* served as outgroups. Values of likelihood bootstrap support values (≥70%, left) and Bayesian significant posterior probabilities (≥0.97, right) are indicated above the nodes. New species and new sequences are in bold. New species are shaded in a red color.

ML and BI analysis of each dataset yielded their own similar tree topologies and therefore the trees inferred from the ML analyses for each dataset are, respectively, shown (Figure 1, Figure 2, Figure 3, Figure 4, Figure 5 and Figure 6). The proposed new species, *R. sinodelica*, is resolved as an independent clade in both ITS (Dataset I) and 3-locus (ITS-nrLSU-rpb2, Dataset II) trees of subgen. *Brevipes*, and is clearly separated from known species (Figure 1 and Figure 2).

The four proposed new species, *R. brevicostata*, *R. demirimosa*, *R. dongyaensis*, and *R. parafluvialis*, are supported by both of the ITS tree (Dataset III) and 3-locus (ITS-nrLSU-rpb2) tree (Dataset IV) of subgen. *Heterophyllidia* (Figure 3 and Figure 4); the erection of the other four proposed new species are supported by the ITS tree (Dataset V) and 3-locus (ITS-rpb2-tef1) tree (Dataset VI) of subgen. *Russula*, *viz. R. liuboanum*, *R. puxianensis*, *R. rubrolivacea*, and *R. sinocurtipes* (Figure 5 and Figure 6).

### 3.2. Taxonomy

**Figure 7:**
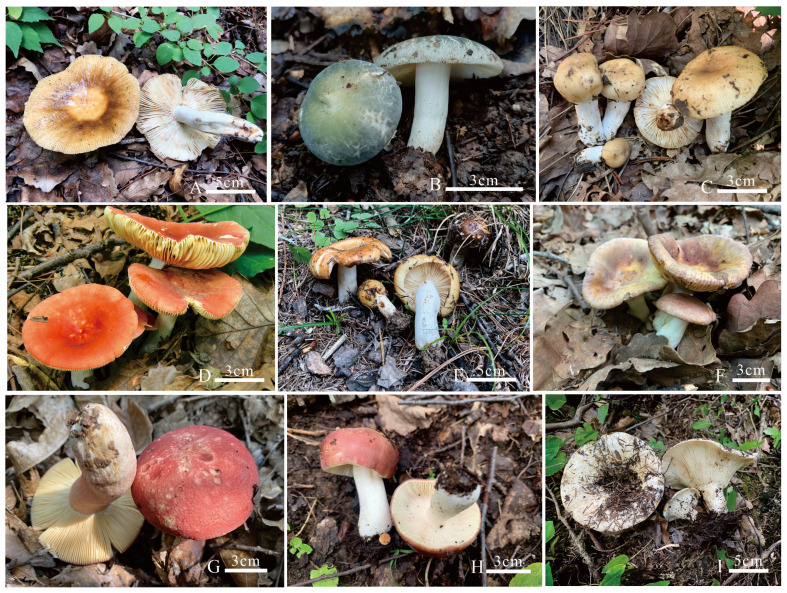
Basidiocarps in the field. (**A**) *Russula brevicostata* (BJTC FM715, holotype); (**B**) *Russula demirimosa* (BJTC FM1006, holotype); (**C**) *Russula dongyaensis* (BJTC FM1753, holotype); (**D**) *Russula liuboanum* (BJTC FM3439, holotype); (**E**) *Russula parafluvialis* (BJTC FM627, holotype); (**F**) *Russula puxianensis* (BJTC FM1868, holotype); (**G**) *Russula rubrolivacea* (BJTC FM1821, holotype); (**H**) *Russula sinocurtipes* (BJTC FM2493, holotype); (**I**) *Russula sinodelica* (BJTC FM1945, holotype).

**Figure 8:**
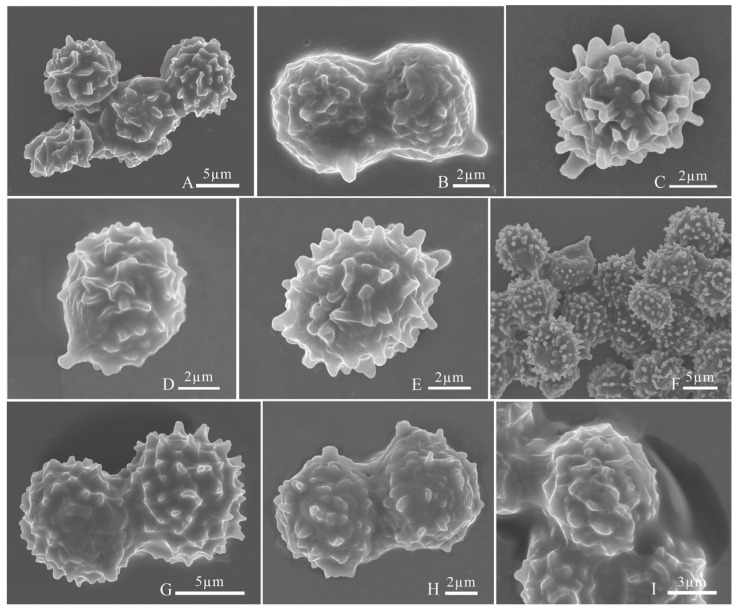
Basidiospores under scanning electron microscope. (**A**) *Russula brevicostata* (BJTC FM715, holotype); (**B**) *Russula demirimosa* (BJTC FM1006, holotype); (**C**) *Russula dongyaensis* (BJTC FM1753, holotype); (**D**) *Russula liuboanum* (BJTC FM3439, holotype); (**E**) *Russula parafluvialis* (BJTC FM627, holotype); (**F**) *Russula puxianensis* (BJTC FM1868, holotype); (**G**) *Russula rubrolivacea* (BJTC FM1821, holotype); (**H**) *Russula sinocurtipes* (BJTC FM2493, holotype); (**I**) *Russula sinodelica* (BJTC FM1945, holotype).

**Figure 9:**
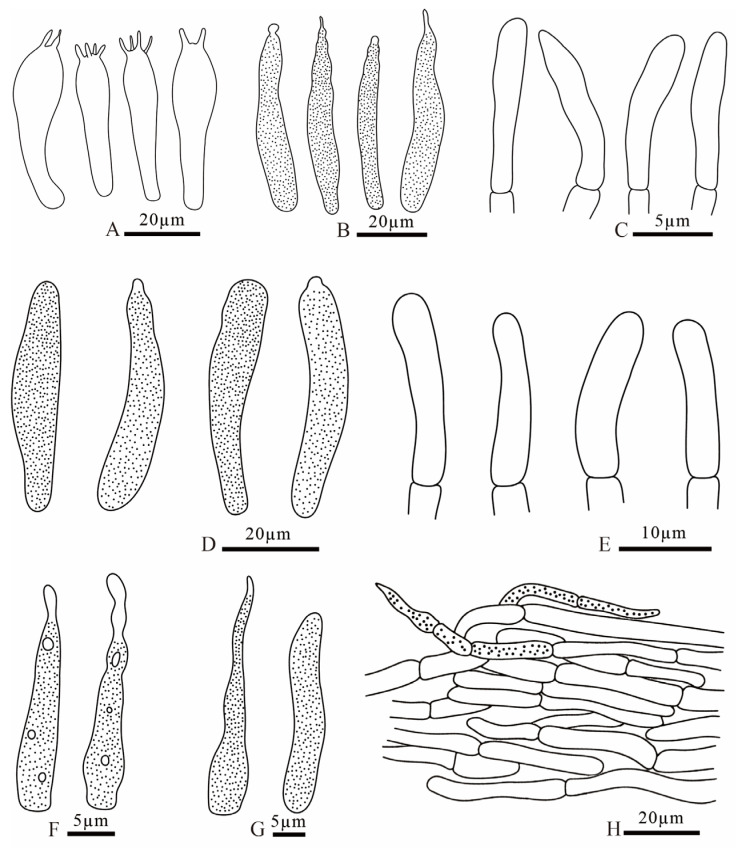
*Russula brevicostata* (FANM1783, holotype) (**A**) basidia; (**B**) pleurocystidia; (**C**) terminal cells of pileipellis; (**D**) cheilocystidia; (**E**) terminal cells of stipitipellis; (**F**) pileocystidia; (**G**) caulocystidia; (**H**) pileipellis.

***Russula brevicostata* L. Fan and H.Y. Fu, Figure 7A, Figure 8A and Figure 9**.

*Mycobank*—MB861771.

*Etymology*—refers to the short ridge ornamentation of spores.

*Holotype*—CHINA. Shanxi Province: Jincheng City, Qinshui County, Lishan Mountains, Zhongcun Forest Farm, 1651 m, 22 August 2019, on the ground in the mix-forest dominated by *Quercus wutaishansea and Pinus tabuliformis*, collected by C. Yang YCM031 (BJTC FM715).

*Description*—*Basidiocarps* medium to large-sized. *Pileus* 4.2–12 cm in diam, at first flat hemispheric to hemispheric, then expanding to flat, applanate, often depressed at center; moderately to strongly tuberculate-striate up to 1/3 to 1/2 pileus radius when mature; surface dull, viscid when wet, pale yellow (#fff3b6), yellow brown (#eac200) or straw yellow (#9e8300). *Lamellae* adnate, lamellulae absent or rare, furcation absent, white (#ffffeb), turning pale yellow (#ffd784) when bruised, edge entire, concolorous. *Stipe* 7–12 × 2.3–4 cm, hollow, with internally bamboo-like septate cavities, nearly equal in thickness, slightly tapered at the base, white (#ffffeb) to cream (#ffd784), surface with longitudinal rugulose with age, turning pale brown (#ffc652) when bruised. *Context* 0.3–0.4 cm thick at pileus center, white (#ffffeb). *Odor* not distinctive. *Taste* not distinctive. *Spore print* cream color.

*Basidiospores* [60/4/2] 7.2–9.6(10.2) × (6.8)7.1–9.3(9.8) μm (Q = 1.04–1.2, Qm = 1.12 ± 0.08), subglobose to globose, ornamentation short ridged or rarely subreticulate, 0.7–1.6 (1.9) μm high, amyloid; *hilar appendix* 1.3–2.5 μm, amyloid. *Basidia* (34.8)38–50.9(54.6) × 10.9–14.9 μm, clavate, 2–4-spored, sterigmata 3.3–7.9 μm long, hyaline in 5% KOH. *Pleurocystidia* 51.1–62.9(67.3) × 8–11.7(12.7) μm, cylindrical-fusiform, apex occasionally minutely papillate or elongate, contents granular, brownish yellow in sulphovanillin. *Cheilocystidia* 40–61.8 × 6.9–12.7 μm, clavate-fusiform to cylindrical, apex papillate, contents granular. *Lamellar trama* irregular. *Subhymenium* 19.1–23.7 μm thick, composed of sphaerocytes and hyphae. *Pileipellis* metachromatic in Cresyl blue, two-layered, *superpellis* an ixocutis, 100.9–126.1 μm thick, composed of hyphae, *terminal cells* 9.8–13.8 × 3–3.9 μm, cylindrical-clavate with occasional subglobose apical swellings (4–5 μm diam); *subpellis* an ixocutis, 190.5–235 μm thick, pigmentation reduced. *Pileocystidia* 15.7–33.7 × 2.6–6.6 μm, flask-shaped, apex aculeate, basal cells inflated, containing granular inclusions. *Terminal cells of stipitipellis*, clavate. *Caulocystidia* (33)35.2–40.5(44.8) × 3.3–6.6 μm, clavate, flask-shaped, containing granular inclusions. *Clamp connections* absent.

*Habitat and distribution*—Solitary on the ground in a mixed coniferous and broad-leaved forest dominated by *Quercus wutaishansea* and *Pinus tabuliformis*.

*Additional specimens examined*—CHINA. Shanxi Province: Jincheng City, Qinshui County, Tuwo Town, Shangwoquan Village, on the ground in the mix-forest dominated by *Quercus wutaishansea* and *Pinus tabuliformis*, 1150 m, 26 July 2021, collected by N. Mao, J.C. Lv, Li F and J.Z. Cao MNM342 (BJTC FM1783).

*Comments*: *Russula brevicostata* is characterized by its lamellae that is nearly completely absent of both furcation and lamellulae, hollow stipes with internally bamboo-like septate cavities, and spores ornamented with short ridges.

Phylogenetically, *R. brevicostata* is classified in the subgen. *Heterophyllidia* (Figure 3 and Figure 4). *Russula quercicola* from Pakistan sisters to *R. brevicostata*, but it can be differentiated by its spores with nearly complete reticulate ornaments. *Russula fluvialis* (Figure 3) and *R. parafluvialis* (Figure 3 and Figure 4), another new species described in this paper, are also related and highly similar to the present species. Of them, *R. fluvialis* is distinguished from *R. brevicostata* by its conifer habitat and different phylogenetic position [50,51]; *R. parafluvialis* by its frequent furcations of lamellae and spores ornamented with isolated warts (this paper).

**Figure 10:**
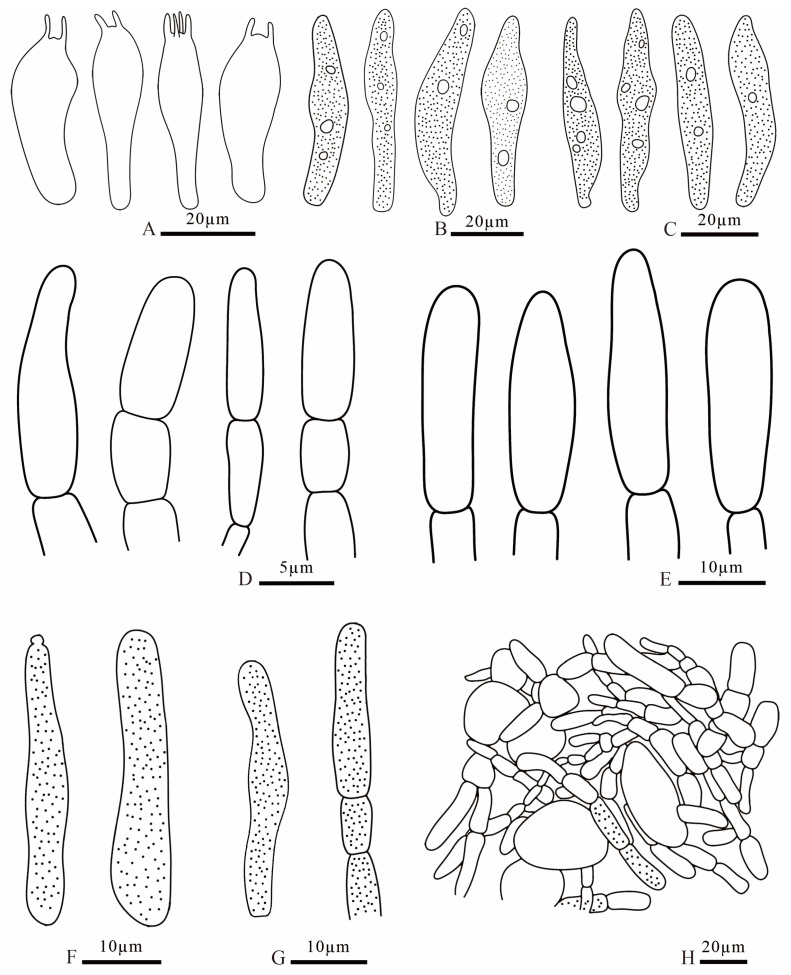
*Russula demirimosa* (FANM1006, holotype). (**A**). basidia; (**B**). pleurocystidia; (**C**). cheilocystidia; (**D**). terminal cells of pileipellis; (**E**). terminal cells of stipitipellis; (**F**). caulocystidia; (**G**). pileocystidia; (**H**). pileipellis.

***Russula demirimosa* L. Fan and H.Y. Fu, Figure 7B, Figure 8B and Figure 10.**

Mycobank—MB861772.

Etymology—refers to the partial cracking of pileal surface.

Holotype—CHINA. Shanxi Province: Jincheng City, Qinshui County, Li Mountains, Zhongcun Forest Farm, 1680 m, 24 August 2020, on the ground in the mix-forest dominated by *Quercus* sp. and *Pinus* sp., collected by J.Z. Cao LH1146 (BJTC FM1006).

Description—*Basidiocarps* small-sized. *Pileus* 3.4–5 cm in diam, flat-hemispheric to applanate, slightly depressed with a recurved margin, partly cracked when mature, surface dry, slightly viscid when wet, dark green (#05af70), gradually transitioning to dull yellowish-green (#7fc806) at the disc, margin whitish-green at maturity. *Lamellae* adnate, dense, lamellulae and furcations absent, white (#ffffeb), aging to cream color (#ffdd35). *Stipe* 4–4.2 × 1–1.2 cm, hollow, cylindrical, tapering basally, cream-colored (#ffdd35), surface longitudinally rugulose with age. *Context* 0.3–1 mm thick, yellowish-white (#ffd784). *Odor* not distinctive. *Taste* not distinctive. *Spore print* cream color.

*Basidiospores* [60/2/1] (5.2)5.7–6.7(7.3) × (4.3)5.0–5.7(5.7) µm, Q = (1.04)1.09–1.29(1.3), Q_m_ = 1.19 ± 0.1, subglobose to broadly ellipsoid, ornamentation of isolated fine warts, 0.7–1.1 µm high, amyloid; *hilar appendix* 0.7–1.1 µm long, amyloid. *Basidia* 31.1–39.8 × 8.3–11.9 µm, clavate, 2–4-spored, hyaline to pale yellow in 5% KOH. *Lamellar trama* dominated by sphaerocytes. *Subhymenium* 30.7–47.8 μm thick, composed of sphaerocytes and hyphae. *Pleurocystidia* (51.5)52.5–66.7(68.5) × 9.3–13.2(15.5) µm, fusiform to flask-shaped, occasionally with apical protrusions, containing refractive inclusions, negative to sulphovanillin. *Cheilocystidia* (47.2)49.1–60.4(62.6) × (7.6)8.6–11.5 µm, morphologically similar to pleurocystidia but smaller. *Pileipellis* a loosely interwoven ixocutis, metachromatic in Cresyl blue; hyphae 3–4 µm wide, slightly thick-walled, yellow-pigmented; *terminal cells* (9.6)11.3–15.5 × 2.6–3.7 µm, cylindrical, sometimes partly inflated, up to 5 µm wide, apically obtuse. *Pileocystidia* (27.6)32.7–33.5(37.9) × 5.4–7.1 µm, containing granular inclusions. *Terminal cells of stipitipellis*, clavate. *Caulocystidia* (32.8)35.2–40.9(42.8) × 5.7–8.0(8.3) µm, clavate, containing granular matter. *Clamp connections* absent.

Habitat—Solitary or scattered on the ground in mixed forests dominated by *Quercus wutaishansea* and *Pinus* sp.

Comments: *Russula demirimosa* is characterized by relatively small basidiocarps, gray-green and partly cracked pileus, spores ornamented with isolated fine warts and fusiform to flask-shaped cheilocystidia.

Phylogenetically, *R. demirimosa* is classified in the subgen. *Heterophyllidia* (Figure 3 and Figure 4) and sisters to *R. swatica* from Pakistan (Figure 3), but *R. swatica* can be differed by its gray green pileus lacking cracks [52]. There are five other species related to the present species, including *R. atroglauca, R. faustiana, R. galochroa, R. subterfurcata*, and *R. shawarensis* (Figure 3). However, *R. atroglauca* and *R. shawarensis* have no cracks on pileus surface; *R. faustiana* exhibits a pale ochraceous pileus with rust-brown maculae or striations, and spore ornamentation featuring cristate ridges and zebroid patterns; *R. subterfurcata* and *R. galochroa* possess white pileus, more or less with ocher, lilac, gray-olive shades, and furcated lamellae (abundant in *R. subterfurcata*, sporadic in *R. galochroa*) [53,54,55,56,57]. All of which distinguish these five species from the present species.

**Figure 11:**
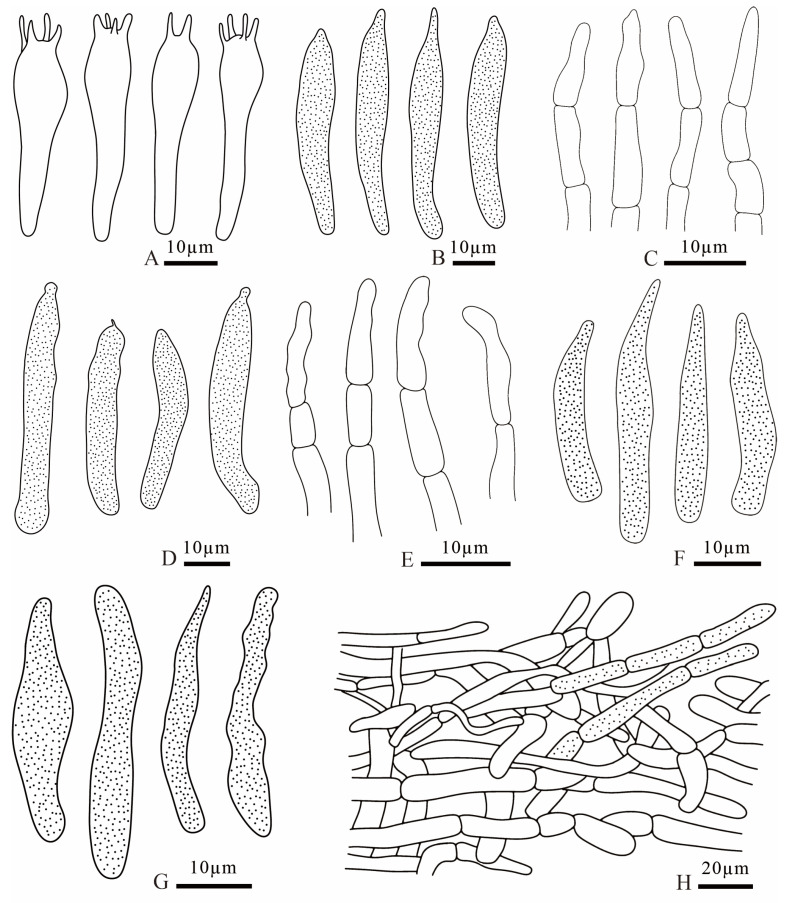
*Russula dongyaensis* (FANM1718, holotype) (**A**) basidia; (**B**) cheilocystidia; (**C**) terminal cells of pileipellis; (**D**) pleurocystidia; (**E**) terminal cells of stipitipellis; (**F**) pileocystidia; (**G**) caulocystidia; (**H**) pileipellis.

***Russula dongyaensis* L. Fan and H.Y. Fu, Figure 7C, Figure 8C and Figure 11.**

Mycobank—MB861773.

Etymology—*dongya*, Chinese, means East Asia, refers to the region where the species is distributed.

Holotype—CHINA. Shanxi Province: Jincheng City, Qinshui County, Li Mountains, Zhongcun Forest Farm, 1640 m, 25 July 2021, on the ground in the forest dominated by *Quercus wutaishansea*, collected by N. Mao, J.C. Lv, Li F and J.Z. Cao MNM355 (BJTC FM1753).

Description—*Basidiocarps* small to medium-sized. *Pileus* 3.5–6 cm diam, at first flat-hemispheric with incurved margin, then expanding to flat, applanate, often depressed at the center when mature; tuberculate-striate up to 1/3 pileus radius, surface slightly viscid when wet, pale yellow (#fff0d2) at first, tinged with pale brown (#ffd456), yellowish brown (#d49f00) to straw color when mature, generally darker at center, *Lamellae* adnate, white (#ffffeb), furcations present, especially near the stipe, lamellulae absent or rare. *Stipe* 5.9–7 × 1.7–2.3 cm, hollow, subequal in thickness, occasionally slightly swollen medially, white (#ffffeb), surface longitudinally striated, slightly turning pale brown (#ffe18a) when bruised. *Context* 0.14–0.3 cm thick at pileus center, white (#ffffeb). *Odor* not distinctive. *Taste* not distinctive. *Spore print* white.

*Basidiospores* [90/10/2] (5.9)6.2–7.8 × (5.2)6.3–5.9(6.4) μm (Q = 1.01–1.5, Qm = 1.25 ± 0.25), globose to ellipsoid, ornamentation shortly cylindrical, apex obtuse or truncate, 0.5–1 μm high, occasionally fused into short ridges or larger warts, amyloid; hilar region with reduced ornamentation; *hilar appendix* 1–2.4 μm, weakly amyloid. *Basidia* (30.2)35.7–46.4(51.6) × 10–11.4(12.4) μm, clavate, 2–4-spored, sterigmata 3.1–7.5 μm long, hyaline in 5% KOH. *Pleurocystidia* (40.7)43.1–55.5(58.4) × 8.1–11.5(13.1) μm, fusiform to elongate-clavate, containing granular inclusions, slightly brownish yellow in sulphovanillin. *Cheilocystidia* 46.2–67.3(69.5) × 8–12.4 μm, clavate-ventricose, with apical prolongations up to 4 μm in length, containing granular inclusions. *Lamellar trama* irregular. *Subhymenium* 19–32.9 μm thick, composed of sphaerocytes and hyphae. *Pileipellis* metachromatic in Cresyl blue, two-layered, *suprapellis* ixocutis, 24.4–53.7 μm thick, composed of hyphae (2–4.4 μm wide), hyaline in 5% KOH, *terminal cells* 5.1–15.9(17.8) × 2.2–4.6 μm, clavate to ventricose, hyaline; *subpellis* cutis, 60.6–79.9 μm thick. *Pileocystidia* (14.7)16.5–39.3(42.5) × 3.2–5.9 μm, fusiform to ventricose, containing granular inclusions. *Terminal cells of stipitipellis* subclavate to clavate. *Caulocystidia* (24.4)28.2–38.9 × 3.4–6.5 μm, fusiform to lageniform, containing granular inclusions. *Clamp connections* absent.

Habitat and distribution—Solitary or scattered on the ground in a broad-leaved forest dominated by *Quercus wutaishansea*.

Additional specimens examined—CHINA. Shanxi Province: Changzhi City, Qinyuan County, Lingkong Mountains, Shengshou Temple, 1510 m, 24 July 2021, on the ground in the mix-forest dominated by *Quercus* sp. and *Pinus* sp., collected by N. Mao, J.C. Lv, Li F and J.Z. Cao LJC008 (BJTC FM1718).

Comments: *Russula dongyaensis* is characterized by its straw-colored pileus, basidiospores ornamented with short cylindrical warts with obtuse or truncate apex, and generally clavate to ventricose cystidia. Phylogenetically, *R. dongyaensis* is classified in the subgen. *Heterophyllidia* (Figure 3 and Figure 4) and closely related to *R. amerorecondita* from North America and *R. recondita* (Figure 3) from Europe. *Russula amerorecondita* is differed from *R. dongyaensis* by its orange, yellow-orange pileus and larger spores of (8–)8.5–9.6–10.7(–11.84) 9 (6.2–)7.4–8.5–9.5(–10.6) µm [1]; *R. recondite* by its ochraceous, fawn, ochre honey, ochre-gray-bistre pileus with almost always darker at the center, the absence of lamellae furcation, and spores ornamented with mainly isolated spines or warts [58]. Moreover, the North American *R. pectinatoides* is morphologically similar to *R. dongyaensis* as it also has small spores, but it is distinguished by its spore ornaments that are almost completely isolated warts.

**Figure 12:**
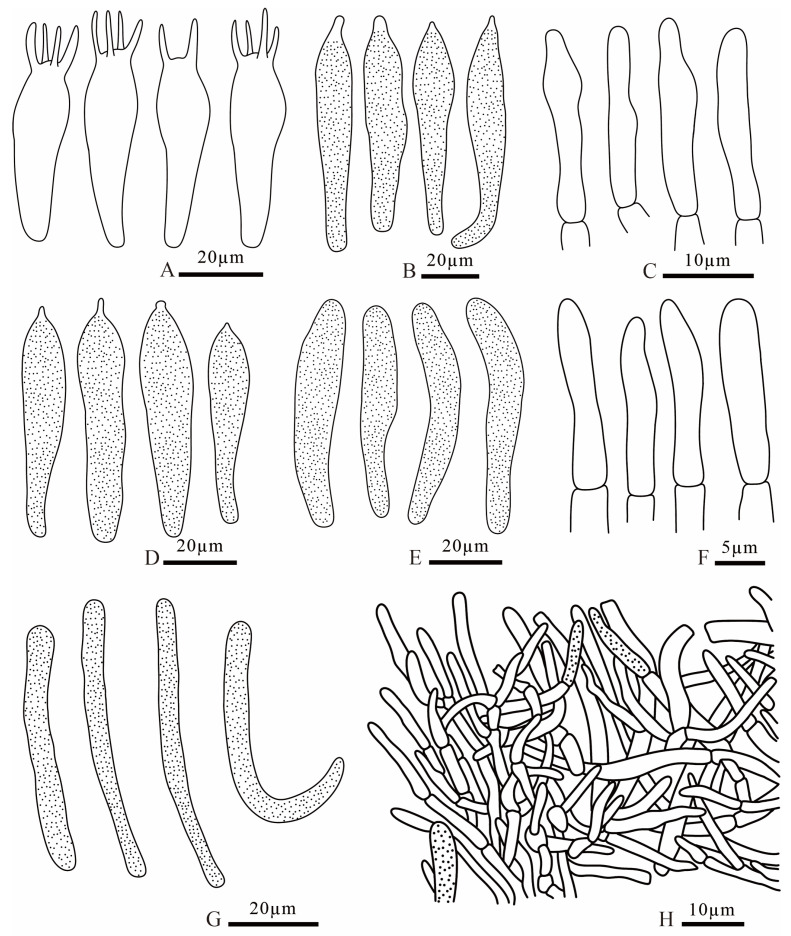
*Russula liuboanum* (FANM3439, holotype) (**A**) basidia; (**B**) pleurocystidia; (**C**) terminal cells of pileipellis; (**D**) cheilocystidia; (**E**) pileocystidia; (**F**) terminal cells of stipitipellis; (**G**) caulocystidia; (**H**) pileipellis.

***Russula liuboanum* L. Fan and H.Y. Fu, Figure 7D, Figure 8D and Figure 12.**

Mycobank—MB861774.

Etymology—refers to honoring Prof. Bo Liu, a Chinese mycologist.

Holotype—CHINA. Shanxi Province: Jincheng City, Lingchuan County, Duohuo Town, on the ground in the forest dominated by *Quercus* sp., 1182 m, 8 August 2023, collected by H.Y. Fu, H.M. Ji and Y. Li MS495 (BJTC FM3439).

Description—*Basidiocarps* medium-sized. *Pileus* 5.2–8.6 cm diam, convex to applanate, slightly depressed at the center, margin very shortly striated, surface viscid to dry, red (#ff4000), peach-red (#ff6b00) to scarlet (#ff974d), often spotted with yellow (#ffdd35) patches. *Lamellae* adnate, dense, lamellulae frequently present, furcations present, white (#ffffeb) at first, creamy (#ffee9c) to off yellowish (#ffe7b6) at maturity, edge entire, concolorous. *Stipe* 7.1–8.9 × 1.8–2.1 cm, hollow, subequal, slightly tapered at the base, white (#ffffeb) to whitish, turning yellowish to pale brown (#ffffb8) when bruised. *Context* 0.3–0.4 cm thick at pileus center, white-cream (#ffffeb). *Odor* indistinct. *Taste* slightly saline. *Spore print* cream color.

*Basidiospores* [90/16/8] 7–8.9 × 6.7–7.8(8.2) μm (Q = 1.01–1.22, Qm = 1.11 ± 0.11), subglobose to broadly ellipsoid, ornamentation verrucouse and shortly ridged, 0.3–0.9 μm high, occasionally interconnected into partly discontinuous reticulum, amyloid; *hilar appendix* (0.6)0.7–1.6(1.7) μm, amyloid. *Basidia* (34.2)36.4–46.3(62) × 10.3–13.6 μm, clavate, 2–4-spored, sterigmata 2.5–9.8 μm long, hyaline in 5% KOH. *Pleurocystidia* 55.9–81.1(87) × 11.5–14(15.4) μm, clavate, apex occasionally elongated (≤7.4 μm), containing granular inclusions, brownish yellow in sulphovanillin. C*heilocystidia* (38.3)42.4–63.7(66.7) × 7.6–14.6 μm, clavate to ventricose, containing granular inclusions. *Lamellar trama* divergent, *Subhymenium* 31.4–34.6 μm thick, composed of sphaerocytes and hyphae. *Pileipellis* metachromatic in Cresyl blue, two-layered: *suprapellis* trichoderm, 41.8–84.4 μm thick, terminal cells 8.5–21.7 × 2.2–4 μm, hyaline, clavate-ventricose; *subpellis* 88.1–184.7 μm thick, composed of interwoven hyphae. *Pileocystidia* (40.5)49.1–70.1(79.7) × (5)6.5–8.5(9.5) μm, clavate to ventricose, containing granular inclusions. *Terminal cells of stipitipellis* subclavate to clavate. *Caulocystidia* (38.1)53.7–64.3(76.4) × (3.5)5.7–6.9(8.6) μm, clavate to ventricose, containing granular inclusions. *Clamp connections* absent.

Habitat—Solitary or scattered on the ground in broad-leaved forests dominated by *Quercus* sp.

Additional specimens examined—CHINA. Shanxi Province: Linfen City, Jiexiu County, Mian Mountains, on the ground in the forest dominated by *Quercus* sp. 1538 m, 7 August 2023, collected by H.Y. Fu, H.M. Ji and Y. Li MS021 (BJTC FM2969), MS025 (BJTC FM2973); ibid, 1393 m, 8 August 2023, collected by H.Y. Fu, H.M. Ji and Y. Li MS085 (BJTC FM3032); ibid, Jincheng City, Qinshui County, Li Mountains, Zhongcun Forest Farm, 1642 m, 10 August 2022, collected by N. Mao, J.C. Lv, Li F and J.Z. Cao MNM585 (BJTC FM2255); ibid, Xiachuan Town, 1821 m, collected by H.Y. Fu, H.M. Ji and Y. Li MS378 (BJTC FM3323); ibid, Lingchuan County, Duohuo Town, 1157 m, 28 August 2023, collected by H.Y. Fu, H.M. Ji and Y. Li MS502 (BJTC FM3446); ibid, 1680 m, 24 August 2020, collected by J.Z. Cao LH1120 (BJTC FM982).

Comments: *Russula liuboanum* is characterized by its red pileus usually spotted with irregular yellow patches, white stipe lacking red flush, and spores ornamented with isolated warts and ridges.

Phylogenetically, *Russula liuboanum* is classified in the subgen. *Russula* (Figure 5 and Figure 6). could be related to the North American *R. pusilla* and the European *R. laeta*, *R. integriformis*, *R. velenovskyi*, and *R. veternosa* (Figure 5). However, *R. pusilla* is morphologically distinguished from *R. liuboanum* by its small basidiomata, light red pileus that is even and absent of yellow spot or patch, and spores ornamentation almost completely reticulated [50,59]; *R. laeta* by its bright yellow to pale ochraceous-yellow pileus, stipe without discoloration when bruised, pale yellow to pale ochraceous spore print, and the absence of caulocystidia [60]; *R. integriformis* by its context turning pale yellow to ochraceous yellow when bruised, pale yellow to yellow spore print, and the absence of caulocystidia [60]. *R. velenovskyi* by its coral red to violet brown pileus lacking spotted yellow patches [61]; *R. veternosa* by its pileus margin with faintly striatations, the absence of lamellulae, stipe no bruising discoloration, pale yellow to pale ochraceous spore print, and the absence of caulocystidia [56].

**Figure 13:**
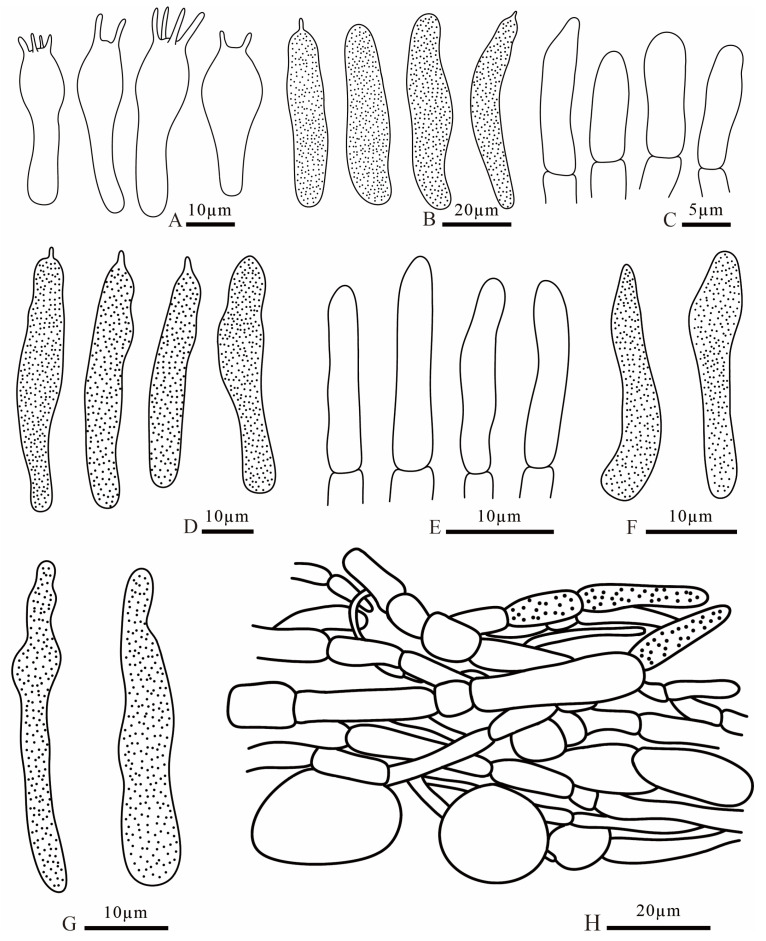
*Russula parafluvialis* (FANM1910, holotype) (**A**) basidia; (**B**) pleurocystidia; (**C**) terminal cells of pileipellis; (**D**) cheilocystidia; (**E**) terminal cells of stipitipellis; (**F**) pileocystidia; (**G**) caulocystidia; (**H**) pileipellis.

***Russula parafluvialis* L. Fan and H.Y. Fu, Figure 7E, Figure 8E and Figure 13.**

Mycobank—MB861775.

Etymology—refers to the similarity to *Russula fluvialis*.

Holotype—CHINA. Shanxi Province: Linfen City, Yicheng County, Shihe Forestry Station, Songshugou, 1907 m, 20 August 2019, on the ground in the mix-forest dominated by *Betula platyphylla, Quercus liaotungensis and Pinus tabulaeformis*, collected by C. Yang YCM003 (BJTC FM627).

Description—*Basidiocarps* small to medium-sized. *Pileus* 2–8 cm diam, flat-hemispheric, hemispherical at first, then flat or convex with a depressed center, moderately to strongly tuberculate-striate up to 2/3 or more of pileus radius when mature, surface viscid to dry, smooth, ochre, beige, light yellow (#e1c606) or straw yellow (#e19006). *Lamellae* adnate, dense, lamellulae present, furcations numerous, white (#ffffeb) to cream (#ffdd35), edge entire, concolorous. *Stipe* 3–12 × 1–2 cm, cylindrical to subcylindrical, slightly tapered or broadly rounded at the base, hollow, white (#ffffeb), smooth, longitudinally rugulose with age, turning pale brown (#fce485) to brown (#faa43b) when bruised. *Context* 2–4 mm thick at pileus center, white (#ffffeb) to yellowish-white (#ffdd35). *Odor* not distinctive. *Taste* not distinctive. *Spore print* cream color.

*Basidiospores* [100/14/7] 7.2–9.3(9.8) × (6.8)7.1–9.6(10.2) μm (Q = 1.01–1.20, Q_m_ = 1.1 ± 0.1), subglobose, occasionally globose, ornamentation of isolated warts, 0.7–1.9 μm high, sparse (2–3 in a 3 μm diam. circle), occasionally connected by low line, amyloid; *hilar appendix* 1.3–2.5 μm, amyloid. *Basidia* (34.8)38–50.9(54.6) × 10.9–14.9 μm, clavate, 2–4-spored, sterigmata 3.3–7.9 μm long, hyaline in 5% KOH. *Pleurocystidia* 51.1–62.9(67.3) × 8–11.7(12.7) μm, cylindrical to subfusiform, apex obtuse, sometimes minutely or slightly long papillate, contents granular, negative to sulphovanillin. *Cheilocystidia* 40–61.8 × 6.9–12.7 μm, clavate to fusiform, sometimes cylindrical, apex papillate, contents granular. *Lamellar trama* bilateral, composed of interwoven hyphae. *Subhymenium* 47.3–60.3 μm thick, composed of sphaerocytes and hyphae. *Pileipellis* not metachromatic in Cresyl blue, two-layered, *suprapellis* an ixocutis, 53.6–60.3 μm thick, terminal cells (9.2)12.1–16.3 × 2.6–3.2 μm, cylindrical to fusiform, cylindrical or mammillate, occasionally inflated into a globose shape; *subpellis* an ixocutis, 58.3–66.5 μm thick. *Pileocystidia* (17.9)19.2–32.8(39.9) × 3–5.9(7.3) μm, subclavate, subfusiform to flask-shaped, with oily granular contents. *Terminal cells of stipitipellis* clavate. *Caulocystidia* 30.4–42.5(47.2) × 4.6–6.8(7.4) μm, subfusiform, flask-shaped, or clavate, contents granular. *Clamp connections* absent.

Habitat and distribution—Scattered or in groups in forest of *Betula platyphylla* or in mixed forests of *B. platyphylla*, *Quercus liaotungensis* and *Pinus tabulaeformis*.

Additional specimens examined—CHINA. Shanxi Province: Linfen City, Huozhou County, Qiliyu Town, 1830 m, 29 July 2021, on the ground in the mix-forest dominated by *B. platyphylla, Quercus liaotungensis and Pinus tabulaeformis*, collected by N. Mao, J.C. Lv, Li F and J.Z. Cao LJC099 (BJTC FM1910); ibid, Lvliang City, Jiaocheng Country, Pangquangou National Nature Reserve, on the ground in the mix-forest dominated by *Quercus* sp. and *Pinus* sp., 1883 m, 18 August 2023, collected by H.Y. Fu, H.M. Ji and Y. Li MS342 (BJTC FM3287); ibid, Jincheng City, Qinshui County, Xiachuan Town, 1782 m, 28 August 2023, collected by H.Y. Fu and H.M. Ji MS387 (BJTC FM3332).

Comments: *Russula parafluvialis* is characterized by its yellowish pileus with long margin tuberculate-striate, lamellae with numerous furcation and spores with isolated warts. Phylogenetically, *R. parafluvialis* is classified in the subgen. *Heterophyllidia* (Figure 3 and Figure 4). *Russula fluvialis* is closely related to our new species (Figure 3); however, *R. fluvialis* is a boreal species occurring in *Picea* spp.-dominated forest, and morphologically it can be differentiated by its pileus with short margin tuberculate-striate up to 1/3 of the radius, the less frequent furcations of lamellae and the smaller spores (7.1–)7.4–7.7–8.1(–8.7) × (6.1–)6.3–6.6–6.9(–7.4) μm with partly connected warts [1]. *Russula brevicostata* and *R. dongyaensis*, the other two new species described in this paper, are also related and highly similar to the present species. Of them, *R. brevicostata* can be differentiated by its different plant host (absolutely associated with *Quercus* spp.), and the ornaments of spores, which is typically short ridges rather than isolated warts; *R. dongyaensis* by its clearly smaller spores of (5.9)6.2–7.8 × (5.2)6.3–5.9(6.4) μm (this paper).

**Figure 14:**
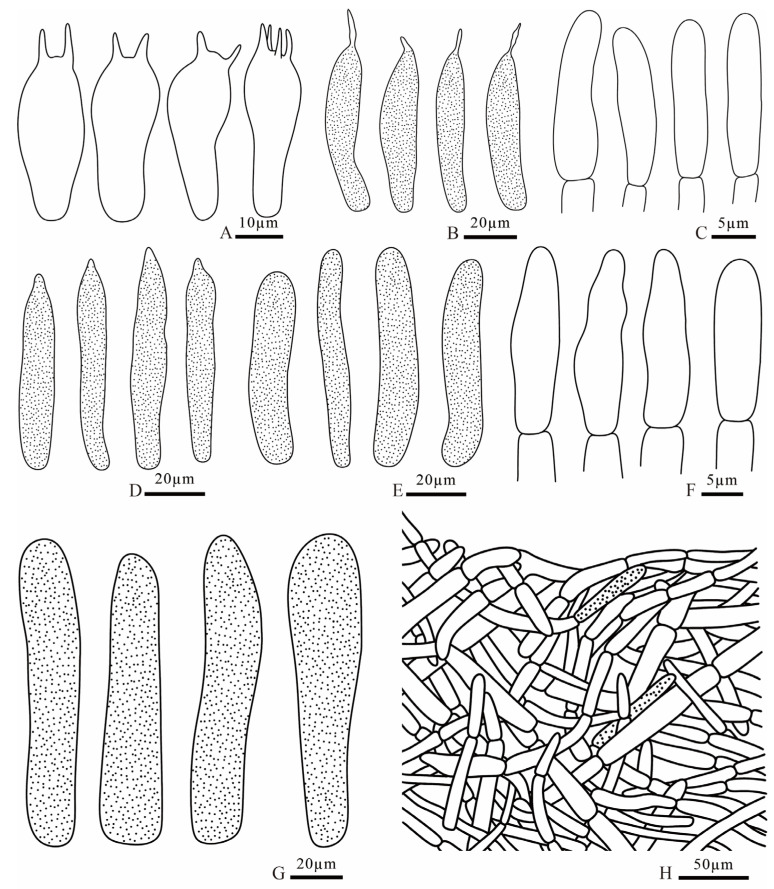
*Russula puxianensis* (FANM1868, holotype) (**A**) basidia; (**B**) pleurocystidia; (**C**) terminal cells of pileipellis; (**D**) cheilocystidia; (**E**) pileocystidia; (**F**) terminal cells of stipitipellis; (**G**) caulocystidia; (**H**) pileipellis.

***Russula puxianensis* L. Fan and H.Y. Fu, Figure 7F, Figure 8F and Figure 14.**

Mycobank—MB861805.

Etymology—refers to the type locality of the new species.

Holotype—CHINA. Shanxi Province: Linfen City, Pu County, Wulu Mountains, on the ground in the forest dominated by *Quercus* sp. 1510 m, 28 July 2021, collected by N. Mao, J.C. Lv, Li F and J.Z. Cao MNM267 (BJTC FM1868).

Description—*Basidiocarps* medium-sized. *Pileus* 4.1–8.5 cm diam, flat-hemispheric to hemispherical at first, then expanding to convex or plano-convex with a depressed center, surface dry, smooth, uneven or spotted, yellow brown (#d1a54a), gray brown (#e6d49b), dark gray brown (#d7ba5e) to dark brown (#a53f2a), margin sometimes tuberculate striated when old and dry. *Lamellae* adnate, dense, lamellulae absent or rare, furcations absent or rare, white (#ffffeb) to cream (#ffdd35), edge entire, concolorous, turning brown (#d28d00) when bruised. *Stipe* 3.5–9 × 1.1–2 cm, cylindrical to subcylindrical, solid, white (#ffffeb), smooth, longitudinally rugulose with age, turning pale brown (#e6d49b) to brown (#d7ba5e) when bruised. *Context* 0.1–0.3 cm thick, white (#ffffeb). *Odor* not distinctive. *Taste* not distinctive. *Spore print* yellow.

*Basidiospores* [80/12/6] (7.3)7.8–10.1(10.9) × (6.4)6.9–9.1(9.6) μm (Q = 1.01–1.3, Qm = 1.15 ± 0.15), globose to broadly ellipsoid, ornamentation echinate, 0.4–0.8 μm high, amyloid; *hilar appendix* (0.6)0.8–1.5(1.8) μm, amyloid. *Basidia* (29.1)31.9–40.8(43.6) × (10.3)11.7–15.1(16.1) μm, ventricose, 2–4-spored, sterigmata 3.6–7.6 μm long, hyaline in 5% KOH. *Pleurocystidia* (49.8)53.2–76.4 × 10.5–13.5 μm, cylindrical to clavate, apically extended with hyaline protrusions (up to 13.9 μm long), containing granular inclusions, nrgative to sulo-vanillin. *Cheilocystidia* (58.5)61.9–73.9(79.4) × (8.8)9.6–12.1 μm, cylindrical to ventricose, containing granular inclusions. *Lamellar trama* irregular, composed of interwoven hyphae and abundant inflated cells of (30.7)40–51.6 × (25.8)27.2–38.8 μm. *Subhymenium* 51–71.8 μm thick, composed of sphaerocytes and hyphae. *Pileipellis* metachromatic in Cresyl blue, two-layered without distinct stratification: *suprapellis* trichoderm, 53.9–88.1 μm thick, terminal cells 11.2–20.1 × 2.2–4.4 μm, hyaline, fusiform to lageniform; *subpellis* Interwoven, 97.1–122 μm thick. *Pileocystidia* (36)36.3–73.1(75.3) × 4.5–8.2 μm, fusiform, containing granular inclusions. *Terminal cells of stipitipellis* subclavate to clavate. *Caulocystidia* (33.2)36.8–58.9(65.5) × 5.6–9.9(11) μm, cylindrical to clavate, containing granular inclusions. *Clamp connections* absent.

Habitat—Solitary on the ground in a broad-leaved forest dominated by *Quercus* sp.

Additional specimens examined—CHINA. Shanxi Province: Linfen City, Pu County, Wulu Mountains, on the ground in the forest dominated by *Quercus* sp. 1510 m, 28 July 2021, collected by N. Mao, J.C. Lv, Li F and J.Z. Cao MNM391 (BJTC FM1899); ibid, alt. 1540 m, 14 August 2022, collected by N. Mao, J.C. Lv, Li F and J.Z. Cao LJC276 (BJTC FM2399); ibid, Linfen City, Jiexiu County, Mian Mountains, 1398 m, 8 August 2023, collected by H.Y. Fu, H.M. Ji and Y. Li. MS086 (BJTC FM3038), MS091 (BJTC FM3038); ibid, Jincheng City, Qinshui County, Li Mountains, Zhongcun Forest Farm, 1642 m, 22 August 2019, collected by C. Yang YCM025 (BJTC FM709).

Comments: *Russula puxianensis* is characterized by its uneven pileus of various shades of brown and gray, but without any red tints, white stipe and spores ornamented with isolated echinates.

Phylogenetically, *Russula puxianensis* is classified in the subgen. *Russula* (Figure 5 and Figure 6) and sisters to *R. heilongjiangensis* (Figure 5), a species described from northeastern China [9]. Morphologically, *Russula heilongjiangensis* possesses a carmine-red pileus and lamellae edge without cheilocystidia, by which it differs from *R. puxianensis*. The European *R. globispora* is sometimes morphologically similar to *R. puxianensis* by its brown pileus with shades of various color; however, the pileus in *R. globispora* has distinct red tints, by which it differs from *R. puxianensis* clearly [62].

**Figure 15:**
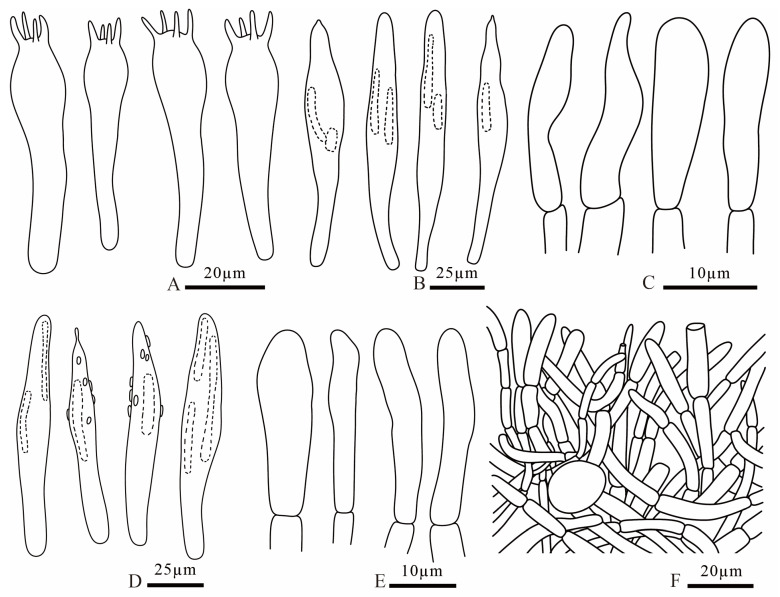
*Russula rubrolivacea* (FANM1821, holotype) (**A**) basidia; (**B**) pleurocystidia; (**C**) terminal cells of pileipellis; (**D**) cheilocystidia; (**E**) terminal cells of stipitipellis; (**F**) pileipellis.

***Russula rubrolivacea* L. Fan and H.Y. Fu, Figure 7G, Figure 8G and Figure 15.**

Mycobank—MB861806.

Etymology—refers to its close relationship to *Russula olivacea* but with red color of cap.

Holotype—CHINA. Shanxi Province: Jincheng City, Qinshui County, Tuwo Town, Shangwoquan Village, on the ground in the forest dominated by *Quercus* sp., 1090 m, 26 July 2021, collected by N. Mao, J.C. Lv, Li F and J.Z. Cao LJC052 (BJTC FM1821).

Description—*Basidiocarps* large-sized. *Pileus* 7.9–10.2 cm diam, hemispherical to applanate, surface dry and usually granulate, red (#b60000), scarlet to dark red (#9d0000). *Lamellae* adnate, lamellulae and furcations present, white (#ffffeb) at first, cream-colored (#ffdd35) to pale yellowish (#ffd784) at maturity, edge entire, concolorous. *Stipe* 8.5–10 × 2–4 cm, cylindrical, solid, white (#ffffeb), sometimes flushed pink (#ffc5c0), surface with longitudinal rugulose with age. *Context* 0.5–0.8 cm thick, white. *Odor* not distinctive. *Taste* slightly burning. *Spore print* cream color.

*Basidiospores* [60/4/3] 8.2–9.5 × 7.7–8.9 μm (Q = 1.02–1.15, Qm = 1.08 ± 0.06), globose to subglobose, ornamentation echinate, 0.4–0.8 μm high, usually interconnected into short ridges or sub-reticulate pattern, amyloid; *hilar appendix* (0.6)1–1.6 μm, amyloid. *Basidia* 48.9–63.4(68.3) × 12.7–14.7 μm, clavate, 2–4-spored, sterigmata 4.4–9.2 μm long, hyaline in 5% KOH. *Pleurocystidia* (90.8)93.1–119.8(121.1) × 10–15.8 μm, ventricose, apically extended with hyaline protrusions (up to 10.3 μm long), containing granular inclusions, negative to sulphovanillin. *Cheilocystidia* (84.6)89.5–108.5(112.1) × (12.6)13.9–15.2(16.7) μm, ventricose, apically extended with hyaline protrusions (up to 5.5 μm long), containing granular inclusions. *Lamellar trama* irregular, composed of interwoven hyphae. *Subhymenium* pseudoparenchymatous, 23.7–31.2 μm thick. *Pileipellis* metachromatic in Cresyl blue, two-layered: *suprapellis* trichoderm, 58.6–93.6 μm thick, hyaline in 5% KOH, terminal cells (10.2)12.1–22.8 × 3.7–5.6(6.6) μm, hyaline, clavate to ventricose; *subpellis* interwoven, 114.2–159.6 μm thick. *Pileocystidia* absent. *Terminal cells of stipitipellis* subclavate to clavate. *Caulocystidia* absent. *Clamp connections* absent.

Habitat—solitary or scattered on the ground in forests dominated by *Quercus* sp.

Additional specimens examined—CHINA. Shanxi Province: Jincheng City, Lingchuan County, Duohuo Town, on the ground in the forest dominated by *Quercus* sp., 1182 m, 27 August 2023, collected by H.Y. Fu, H.M. Ji and Y. Li MS470 (BJTC FM3415); ibid, Qinshui County, Tuwo Town, Shangwoquan Village, on the ground in the mix-forest dominated by *Quercus* sp. and *Pinus* sp., 1090 m, 26 July 2021, collected by N. Mao, J.C. Lv, Li F and J.Z. Cao LJC052-1 (BJTC FM1821-1).

Comments: *Russula rubrolivacea* is characterized by its red pileus surface with fine verrucose projections, white stipe sometimes with flushed pink, spores ornamented with echinate, which are usually interconnected into short ridges or sub-reticulate pattern, and the absence of pileocystidia. Moreover, the specific association with *Quercus acutissima* and *Q. variabilis* might also be unique for this new species.

Phylogenetically, *Russula rubrolivacea* is classified in the subgen. *Russula* (Figure 5 and Figure 6) and sisters to the European *R. olivacea* (Figure 1); however, *R. olivacea* is differentiated from *R. rubrolivacea* by its smooth olivaceous pileus and spores ornamented fine verrucae [63].

**Figure 16:**
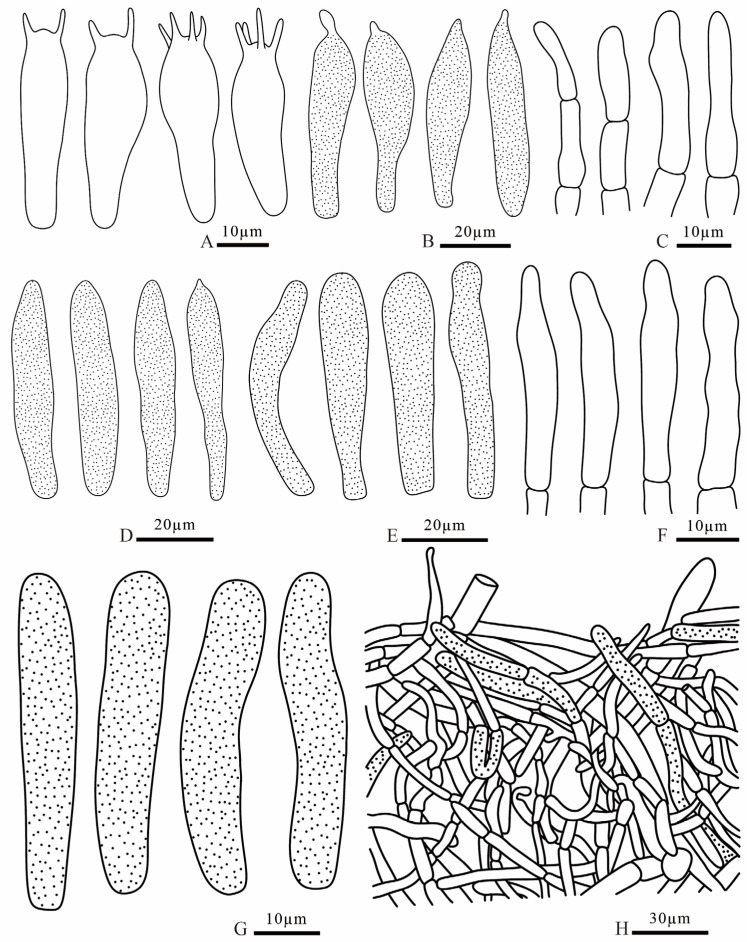
*Russula sinocurtipes* (FANM3291, holotype) (**A**) basidia; (**B**) pleurocystidia; (**C**) terminal cells of pileipellis; (**D**) cheilocystidia; (**E**) pileocystidia; (**F**) terminal cells of stipitipellis; (**G**) caulocystidia; (**H**) pileipellis.

***Russula sinocurtipes* L. Fan and H.Y. Fu, Figure 7H, Figure 8H and Figure 16.**

Mycobank—MB861807.

Etymology—refers to the closely relationship with *Russula curtipes* in phylogeny.

Holotype—CHINA. Shanxi Province: Taiyuan City, Loufan Country, Yunding Mountains, on the ground in the forest dominated by *Quercus* sp., 1824 m, 23 August 2022, collected by J.C. Lv and N. Mao MNM730 (BJTC FM2493).

Description—*Basidiocarps* small to medium-sized. *Pileus* 3.7–6.8 cm diam, flat-hemispheric at first, then pulvinate to applanate; surface dry, yellow brown (#d1614a) when young, wine red (#ea686f) to pink-purple (#f0959a) at maturity, usually with a pale yellow (#ffdd35) center. *Lamellae* adnate, dense, lamellulae absent, furcation numerous, white (#ffffeb) to cream-colored (#ffdd35) at first, yellow (#ffd784) at maturity, edge entire, concolorous. *Stipe* 4–6.4 × 0.8–1.9 cm, hollow, slightly bulbous at the base, white (#ffffeb), smooth, surface with longitudinally rugulose with age. *Context* 0.2–0.4 cm thick at pileus center, white (#ffffeb) to cream (#ffdd35). *Odor* not distinctive. *Taste* faintly salty. *Spore print* yellow.

*Basidiospores* [60/4/2] 6.4–7.7(8.8) × 6–7.9 μm (Q = 1.01–1.16, Qm = 1.08 ± 0.08), subglobose to globose, ornamentation of isolated warts and discontinuous reticulate ridges, 0.4–0.7 μm high, amyloid; *hilar appendix* (0.7)0.9–1.2(1.5) μm, amyloid. *Basidia* 31.3–40.7(42.7) × (9.1)10.7–12.1(13.2) μm, clavate to ventricose, 2–4-spored, sterigmata 3.1–6.9 μm long, hyaline in 5% KOH. *Pleurocystidia* (38.1)40–60 × 7–10.8 μm, clavate to ventricose, containing granular inclusions, slightly gray in sulphovanillin. *Cheilocystidia* (32.1)35.4–57.8(69.6) × 6.1–10.7(13.3) μm, clavate, containing granular inclusions. *Lamellar trama* irregular, composed of hyphae and inflated cells of 8.1–15 μm diam. *Subhymenium* 25.9–30.2 μm thick, cellular. *Pileipellis* non-metachromatic in Cresyl blue, two-layered, *suprapellis* trichoderm, 66.6–84.5 μm thick, *terminal cells* 12.3–30.5 × 1.9–5.7(7.1) μm, hyaline, clavate to lageniform; *subpellis* 72.7–81.9 μm thick, composed of interwoven hyphae. *Pileocystidia* (33.2)36.3–55.2(65.2) × 4.5–8.6 μm, clavate, containing granular inclusions. *Terminal cells of stipitipellis* subclavate to clavate. *Caulocystidia* (31.2)34.1–51.1(54.7) × (3.4)5–8.1(9.1) μm, clavate, containing granular inclusions. *Clamp connections* absent.

Habitat—solitary on the ground in a broad-leaved forest dominated by *Quercus* sp.

Additional specimens examined—CHINA. Shanxi Province: Lvliang City, Jiaocheng Country, Pangquangou National Nature Reserve, on the ground in the mix-forest dominated by Quercus sp. and *Picea asperata* Mast., 2179 m, 18 August 2023, collected by H.Y. Fu, H.M. Ji and Y. Li MS346 (BJTC FM3291).

Comments: *Russula sinocurtipes* is characterized by its wine red to pink-purple pileus usually with a pale-yellow center area, lamellulae absent, basidiospores ornamented with warts and discontinuous reticulate ridges, clavate to ventricose cystidia.

Phylogenetically, *R. sinocurtipes* is classified in the subgen. *Russula* (Figure 5 and Figure 6) and closely related to the European *R. curtipes* and *R. fontqueri* (Figure 1). Morphologically, *R. curtipes* is differentiated from *R. sinocurtipes* by its purple pileus, stout stem up to 1–4 cm in diam, and larger spores up to 7–9.5 × 6.5–7.5 µm [55]; *R. fontqueri* by its multi-colored pileus but lacking purple tints [64].

**Figure 17:**
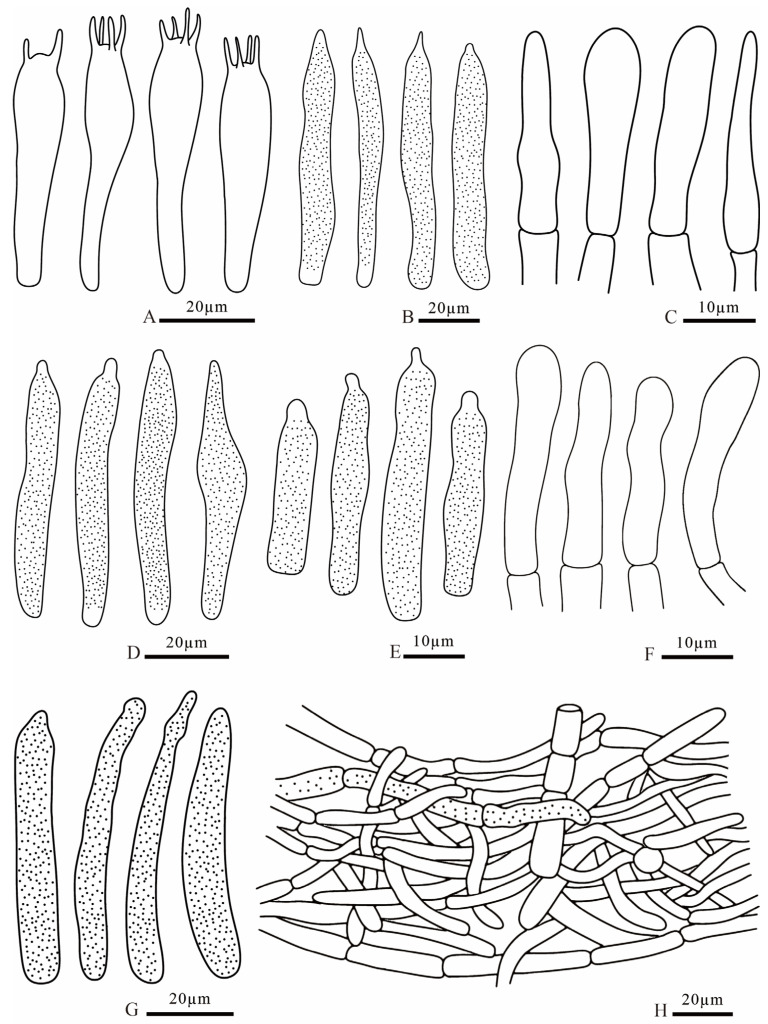
*Russula sinodelicata* (FANM1945, holotype) (**A**) basidia; (**B**) pleurocystidia; (**C**) terminal cells of pileipellis; (**D**) cheilocystidia; (**E**) pileocystidia; (**F**) terminal cells of stipitipellis; (**G**) caulocystidia; (**H**) pileipellis.

***Russula sinodelica* L. Fan and H.Y. Fu, Figure 7I, Figure 8I, and Figure 17.**

Mycobank—MB861808.

Etymology—refers to similarity to *Russula delica* in appearance of basidiocarps.

Holotype—CHINA. Shanxi Province: Lvliang City, Jiaocheng Country, Pangquangou National Nature Reserve, on the ground in the mix-forest dominated by *Quercus* sp. and *Picea asperata* Mast., 1700 m, 30 July 2021, collected by J.C. Lv, N. Mao, Li F and J.Z. Cao CF1119 (BJTC FM1945).

Description—*Basidiocarps* medium to large sized. *Pileus* 5.5–13 cm diam, flat-hemispheric to applanate, depressed at center, margin non-striated, surface slightly viscid to dry, white (#ffffeb), spotted with yellow brown (#d3ac00) patches. *Lamellae* adnate to subdecurrent, dense, lamellulae frequently present in different length, furcations absent, white (#ffffeb) to cream (#ffdd35), edge entire, concolorous. *Stipe* robust, 2.5–5.5 × 1.6–3 cm, solid, subequal, slightly tapering at base; surface white (#ffffeb) to cream (#ffdd35) longitudinal rugulose, but becoming smooth towards the base. *Context* 0.63–1.38 cm thick, white (#ffffeb). *Odor* not distinctive. *Taste* slightly salty. *Spore print* white to cream.

*Basidiospores* [90/6/3] 6.2–8.7 × 5.3–7.5 μm (Q = 1.01–1.22, Qm = 1.11 ± 0.1), subglobose to broadly ellipsoid, ornamentation of isolated warts, or short ridges, 0.5–1.3 μm high, sometimes interconnected into discontinuous reticulum, amyloid, hilar region with reduced ornamentation; *hilar appendix* (0.5)0.7–1.3(1.9) μm, pseudoamyloid. *Basidia* (40.9)43.6–56.2(59.5) × 9–11.7 μm, clavate, 2–4-spored, sterigmata 1.9–7.2 μm long, hyaline in 5% KOH. *Pleurocystidia* (54.8)63.1–85.3 × (6.4)6.8–8.8(9.2) μm, elongated clavate to clavate, medially slightly inflated, apex occasionally mucronate (≤6 μm), containing granular inclusions, gray in sulphovanillin. *Cheilocystidia* 41.4–65.5(70.4) × 5.8–8.1(11.3) μm, clavate-ventricose, containing granular inclusions. *Lamellar trama* irregular, composed of interwoven hyphae, *Subhymenium* 9.3–16.8 μm thick, composed of interwoven hyphae. *Pileipellis* metachromatic in Cresyl blue, two-layered, *suprapellis* ixocutis, 17.2–23.8 μm thick, hyaline in 5% KOH, *terminal cells* 16–30.5(34) × 2.7–5.5(6.4) μm, hyaline, clavate to ventricose; *subpellis* ixocutis, 48–65.5 μm thick. *Pileocystidia* 29.3–44.7(51.8) × 4.6–7.9 μm, clavate to ventricose, containing granular inclusions. *Terminal cells of stipitipellis* clavate. *Caulocystidia* (40.5)48.6–71.8(76) × 5.1–6.6 μm, clavate to lageniform, with granular inclusions. *Clamp connections* absent.

Habitat—Solitary or scattered on the ground in a mixed coniferous and broad-leaved forest dominated by *Quercus liaotungensis* and *Picea asperata.*

Additional specimens examined—CHINA. Shanxi Province: Lvliang City, Jiaocheng Country, Pangquangou National Nature Reserve, on the ground in the mix-forest dominated by *Quercus* sp. and *Picea asperata* Mast., 1900 m, 30 July 2021, collected by J.C. Lv, N. Mao, Li F and J.Z. Cao CF1119 (BJTC FM1965); ibid, Xinzhou City, Fanshi Country, Yantou Town, Erqielan Village, 2204 m, 12 August 2023, collected by H.Y. Fu, H.M. Ji and Y. Li. MS106 (BJTC FM3053).

Comments: *Russula sinodelicata* is characterized by its large basidiomata, white pileus with brown spots, adnate to subdecurrent lamellae, small basidiospores, and the unique association with *Picea* spp.

*Russula sinodelicata* is highly similar to the North American native *R. brevipes* and the European native *R. delica* as all of the three species possesses large and white basidiocarps. However, *R. brevipes* is differentiated from *R. sinodelicata* by its whitish to dull ivory pileus with ochraceous tones, decurrent lamellae showing white when young but becoming pale yellow to buff with age, and relatively large spores ((8.3)8.5–10.4(12.0) × (6.8)7.0–8.8(9.0) μm) [65]; *R. delica* by its larger spores up to 8–12 × 7–9 μm and association with *Pinus* spp. [66].

Phylogenetically, *R. sinodelicata* is classified in the subgen. *Brevipes* (Figure 1 and Figure 2) and related to *R. australis*, *R. byssina*, *Russula pumicoidea* (Figure 1), and *R. sinuata*, yet shows morphological distinctions. *Russula byssina* possesses a creamy white to pale brownish-orange pileus and adnate to slightly decurrent lamellae [67]; *R. sinuata*, *R. australis*, and *R. pumicoidea* are all sequestrate fungi lacking typical agaricoid basidiocarps, all of which are completely different from those in *R. sinodelicata* [68,69].

## 4. Discussion

The genus *Russula* is characterized by the brightly colored caps, brittle flesh, and absence of latex. Distinguishing *Russula* species based solely on morphological traits is challenging; thus, current species identification within *Russula* primarily relies on phylogenetic analysis based on ITS sequence or/and multi-locus combined data, especially differentiating and interpreting closely related species [1,2,5,15,17,23,24,36,50,70,71]. The erection of nine newly named species in this study is well supported by ITS sequence-based and multilocus combined phylogenetic analysis, and belong to three different subgenera: subgen. *Brevipes*, subgen. *Compactae*, and subgen. *Heterophylidia*.

Species of *Russula* subgen. *Brevipes* typically develop medium-large basidiocarps (5–15 cm diam.) featuring staining yellowish-brown pileus, green-tinting lamellae, and short rusty-based stipe, basidiospores with low interconnected warts and cystidia graying in sulphovanillin, occurring strictly in ectomycorrhizal association with Pinaceae or Faga-ceae within temperate to subtropical forests [17,67]. There are many species reported in China, such as *R. brevispora* Y.L. Chen and J.F. Liang, *R. byssina* G.J. Li and C.Y. Deng, *R. cal-lainomarginis* J.F. Liang and J. Song, *R. chloroides* (Krombh.) Bres., *R. cremicolor* G.J. Li and C.Y. Deng, *R. leucocarpa* G.J. Li and C.Y. Deng, *R. luteolamellata* H. Zhou and G.Q. Cheng, R. sub-brevipes J.F. Liang and J. Song [38,40,67,70]. In this study, ITS phylogenetic analyses showed significant support (BS = 97%) for *R. sinodelica*, morphological and phylogenetic results could distinguish this new species well from other known species within this subgenus.

Species of *Russula* subg. *Heterophyllidia* typically have medium to large basidiocarps (4–15 cm diam.) featuring diverse pileus colors (gray, green, purple, or rarely reddish tones) with surfaces often cracking into distinct patches (virescent-type), mild to strongly acrid taste, and white to cream spore prints (rarely ochre), basidiospores with inamyloid or partially amyloid suprahilar spots and ornamentation of warts fused into short/long chains, abundant gloeocystidia (typically mucronate to obtuse-rounded), pileipellis with inflated hyphal terminations (ellipsoid/globose cells + attenuated terminal cells), and absence of primordial hyphae, occurring in ectomycorrhizal association with Fagaceae, Pinaceae, or Dipterocarpaceae across temperate to tropical forests [2,56,70]. In this study, ITS phylogenetic analyses showed significant support for *R. brevicostata* (BS = 99%), *R. demirimosa* (BS = 100%), *R. dongyaensis* (BS = 100%) and *R. parafluvialis* (BS = 99%), morphological and phylogenetic results could distinguish these new species from each other and from other known species within this subgenus.

Species of *Russula* subgen. *Russula* typically have small to medium-sized basidiocarps (3–10 cm diam.) featuring brightly colored pileus (red, purple, green, or yellow) with smooth or striate margins, brittle context turning yellowish-red when bruised, and pink-ish-white stipe often developing rusty spots, basidiospores with amyloid suprahilar spots and ornamentation of isolated to partially connected warts (0.3–1.0 µm high), abundant hymenial cystidia with variable apex shapes (capitate, moniliform, or appendiculate) re-acting intensely in sulphovanillin, and a pileipellis lacking well-differentiated dermato-cystidia, occurring exclusively in ectomycorrhizal association with broad-leaved trees (e.g., Fagaceae, Betulaceae) across boreal to Mediterranean forests [2,56,72]. In this study, ITS phylogenetic analyses showed significant support for *R. liuboanum* (BS = 100%), *R. puxianensis* (BS = 100%), *R. rubrolivacea* (BS = 100%) and *R. sinocurtipes* (BS = 100%), with other species in this subgenus, while morphological and phylogenetic results could distinguish these new species from each other and from other known species within this sub-genus.

Moreover, the ECM genus *Russula* received less taxonomic treatment in Shanxi Prov-ince before this study, and total 25 species had been recorded from this area, including *R. aeruginea*, *R. atroglauca*, *R. aurea*, *R. azurea*, *R. cessans*, *R. delica*, *R. exalbicans*, *R. yanoxantha*, *R. gracillima*, *R. integra*, *R. laricina*, *R. lilacea*, *R. lutea*, *R. nauseosa*, *R. odorata*, *R. pallidospora*, *R. pascua*, *R. persicina*, *R. renidens*, *R. sanguinea*, *R. subfoetens*, *R. vesca*, *R. vinosa*, *R. virescens*, and *R. xerampelina* [71,73,74]. However, our study based on both morphological and mo-lecular evidences revealed that there were at least 65 species occurring in this area in-cluding the nine new species described in this study, which indicated Shanxi probably has a high diversity of *Russula* species actually (unpublished). It is therefore clear that more efforts could be needed for clarifying the species diversity of Russula of this important geographic region.

## 5. Conclusions

Based on integrative morphological and molecular evidence, nine new species of *Russula* distributed across three subgenera were identified and described herein. The newly established species are *R. sinodelica* (subg. *Brevipes*); *R. brevicostata*, *R. demirimosa*, *R. dongyaensis*, and *R. parafluvialis* (subg. *Heterophyllidia*); and *R. liuboanum*, *R. puxianensis*, *R. rubrolivacea*, and *R. sinocurtipes* (subg. *Russula*).

## Data Availability

The newly generated sequences have been uploaded to GenBank with accession numbers in the article and Supplementary Materials. The original contributions presented in this study are included in the article/Supplementary Materials. Further inquiries can be directed to the corresponding authors.

## Supplementary Materials

The following supporting information can be downloaded at: https://www.mdpi.com/article/10.3390/jof12010078/s1. Supplement 1. Samples used for ITS and multi-locus phylogenetic analysis (*Russula* subgen. *brevipes*) and their GenBank accession numbers. Sequences newly generated in this study are in bold. Holotype specimen is marked. Supplement 2. Samples used for ITS phylogenetic analysis (*Russula* subgen. *heterophylidia*) and their GenBank accession numbers. Sequences newly generated in this study are in bold. Holotype specimen is marked. Supplement 3. Samples used for multi-locus phylogenetic analysis (*Russula* subgen. *heterophylidia*) and their GenBank accession numbers. Sequences newly generated in this study are in bold. Holotype specimen is marked. Supplement 4. Samples used for ITS phylogenetic analysis (*Russula* subgen. *russula*) and their GenBank accession numbers. Sequences newly generated in this study are in bold. Holotype specimen is marked. Supplement 5. Samples used for multi-locus phylogenetic analysis (*Russula* subgen. *russula*) and their GenBank accession numbers. Sequences newly generated in this study are in bold. Holotype specimen is marked.
