# Supplementary material for: Morphological and Phylogenetic Evidence Reveal Nine New Species of Russula (Russulaceae, Russulales) from Shanxi Province, North China
Source: J Fungi (Basel). 2026 Jan 22;12(1):78. doi: 10.3390/jof12010078 (PMC12843102; doi:10.3390/jof12010078)
Supplement: Supplementary file 1 [file jof-12-00078-s001.zip › Supplement 1.pdf]

Supplement 1. Samples used for ITS and multi-locus phylogenetic analysis (*Russula* subgen. *brevipes*) and their GenBank accession numbers. Sequences newly generated in this study are in bold. Holotype specimen is marked.

Species	Voucher	Locality	GenBank accession No.		
			ITS	LSU	*rpb2*
Russula mustelina	FH12226	Germany	KT934005	KT933866	KT933937
Russula vesca	BPL284	USA	KT933978	KT933839	KT933910
Russula albocarpa	HGASMF009910	China	MN648948	-	-
Russula brevipes	QHU20108		OM970921	-	-
Russula brevipes	SMI329		FJ845429	-	-
Russula byssina	HGAS MF009907	China	NR 169999	-	-
Russula laevis	JR4016	Finland	MN130091	MN130128	-
Russula luteolamellata	BJTC 0534	China	ON921139	ON908461	-
Russula luteolamellata	BJTC T2201	China	ON921140	ON908462	-
Russula aff chloroides	FH12273	Belgium	KT934015	KT933876	-
Russula aff delica	BB 12086	Italy	-	KU237594	KU237879
Russula aff pallidospora	MPG13608	Spain	-	KU237580	KU237866
Russula australis	JAC10732	New Zealand	MW683746	MW683616	-
Russula brevipes	BB 06508	Mexico	-	KU237479	KU237765
Russula brevipes var acrior	JMP0058	USA	EU819422	-	-
Russula byssina	HGAS MF009913	China	MN648950	-	-
Russula byssina	HGAS MF009921	China	MN648949	-	-
Russula callainomarginis	Li16091020	China	MH911606	-	-
Russula callainomarginis	Li15073109	China	MH911605	-	-
Russula callainomarginis	RITF2639	China	MH286463	MH286468	MH911624
Russula camarophylla	PAM01081108	China	DQ421982	-	-
Russula cf brevipes	BB 06441	Mexico	-	KU237483	KU237769
Russula cf delica	SA07210	Slovakia	-	KU237600	KU237885
Russula chloroides	UBCF20353	Canada	KC581331	KC581331	KT933947
Russula delica	FH12272	Belgium	KF432955	KR364224	KR364340
Russula delicinae	BB 06476	Mexico	-	KU237484	KU237770
Russula herrerae	BB 06532	Mexico	-	KU237486	KU237772
Russula marangania	MEL2293694	Australia	EU019930	EU019930	-
Russula pallidospora	JV02218	Sweden	DQ422032	DQ422032	-
Russula pumicoidea	Trappe14771	Australia	EU019931	EU019931	-
Russula sinodelica	**BJTC FM3053**	**China**	**PX778477**	**PX778507**	**PX789036**
Russula sinodelica	**BJTC FM1965**	**China**	**PX778478**	**-**	**-**
Russula sinodelica	**BJTC FM1945 holotype**	**China**	**PX778476**	**PX778506**	**PX789035**
Russula sinuata	H4755	Australia	EU019943	-	-
Russula subbrevipes	RITF3136	China	MH286460	MH286465	MH911625
Russula subbrevipes	RITF3136	China	MH286460	-	MH911625
Russula subbrevipes	RITF2946	China	MH286462	MH286467	-
Russula subbrevipes	RITF3002	China	MH286461	MH286466	-
Russula vesicatoria	BB 07034	USA	-	KU237599	KU237884
