# Supplementary material for: Morphological and Phylogenetic Evidence Reveal Nine New Species of Russula (Russulaceae, Russulales) from Shanxi Province, North China
Source: J Fungi (Basel). 2026 Jan 22;12(1):78. doi: 10.3390/jof12010078 (PMC12843102; doi:10.3390/jof12010078)
Supplement: Supplementary file 1 [file jof-12-00078-s001.zip › Supplement 2.pdf]

Supplement 2. Samples used for ITS phylogenetic analysis (*Russula* subgen. *heterophylidia*) and their GenBank accession numbers. Sequences newly generated in this study are in bold. Holotype specimen is marked.

Species	Voucher	Locality	GenBank accession No. ITS
Russula aeruginea	69IJ16	Czech Republic	MG680182
Russula aeruginea	DG88	UK	JQ888195
Russula aff. Brunneoannulata	AB57		KR819061
Russula aff. Brunneoannulata	AB56		KR819060
Russula ahmadii	LAH35004	PAK	KT834638
Russula albolutea	RITF2653	China	MT672478
Russula albonigra	SAV F-20197	Slovakia	MW172299
Russula amerorecondita	F (PGA17-017)	USA	MN130066
Russula amerorecondita	SH120828	USA	MN130067
Russula amoenolens	JAC14002	New Zealand	OR348166
Russula amoenolens	JAC9208	New Zealand	MW683721
Russula amoenolens	111	ITA	KJ834593
Russula arvnii	CUH:AM261	IND	KR872619
Russula atroaeruginea	53626	China	JX391967
Russula atrochermesina	RITF6878	China	OR907106
Russula atrochermesina	RITF6460	China	OR907107
Russula atroglauca	HBAU15079	China	MW850413
Russula aureoviridis	RITF4709	China	MW646980
Russula brevicostata	**BJTC FM1783**	**China**	**PX778479**
Russula brevicostata	**BJTC FM715 holotype**	**China**	**PX778480**
Russula catilus	SFC20120827-01	KOR	KX574686
Russula cerolens	001114-28		HQ604835
Russula cerolens	001007-01	Canada	HQ604829
Russula cerolens	760410	USA	KF683922
Russula cf. crustosa	DSL002	Thailand	MT559557
Russula cf. pectinatoides	WTU_F_38909	USA	KF245493
Russula crustosa	BPL265	USA	KT933966
Russula cyanoxantha	FH 12–201	Germany	KR364093
Russula cyanoxantha	UE29.09.2002–2	France	DQ422033
Russula demirimosa	**BJTC FM1006 holotype**	**China**	**PX778481**
Russula dinghuensis	GDGM45244	China	KU863579
Russula dinghuensis	RITF5142	China	MW646982
Russula discoidea	N.K. Zeng4895 (FHMU4847)	China	OP837469
Russula dongyaensis	**BJTC FM1718**	**China**	**PX778483**
Russula dongyaensis	**BJTC FM1753 holotype**	**China**	**PX778482**
Russula echidna	HO 593336	Australia	MN130079
Russula echidna	HO 593335	Australia	MN130081
Russula faustiana	15020	USA	JF908705

Russula faustiana	FH 2011 BT010	Germany	MT738276
Russula floriformis	Corrales1007	Colombia	MT068206
Russula floriformis	Corrales952	Colombia	MT068205
Russula fluvialis	KUO (JR8666)	Finland	MN130084
Russula fluvialis	KUO (JR8313)	Finland	MN130085
Russula foetens	GENT:FH-12-277	DEU	KT934016
Russula foetentoides	K-23	PAK	HE647707
Russula foetentula	160	USA	KJ834626
Russula fragrantissima	108	ITA	KJ834596
Russula fusiformata	K15052703	China	MK049978
Russula fusiformata	RITF6671	China	OR907108
Russula galochroa	FH 2010 BT137	Germany	MT738279
Russula galochroa	FH 2009 BT02	Germany	MT738278
Russula garyensis	F (PGA17-008)	USA	MN130088
Russula garyensis	F (PGA15-910)	USA	MN130089
Russula gelatinosa	K15052626	CHN	MH168575
Russula granulata	161	USA	KJ834625
Russula grata	nl1348	DEU	UDB000344
Russula heterophylla	UE20.08.2004–2	Sweden	DQ422006
Russula ilicis	FU19M	Italy	OQ704262
Russula ilicis	563IC52		AY061682
Russula illota	97	ITA	KJ834605
Russula inamoena	107	ITA	KJ834597
Russula inamoena	109	ITA	KJ834595
Russula indoilicis	UZ-04-18	India	MW547505
Russula indoilicis	UZ-125-21	India	MW725579
Russula insignis	M54		MW355003
Russula insignis	94	FRA	KJ834606
Russula insignis	148	ITA	KJ834553
Russula ionochlora	FH 2010 BT141	Germany	MT738289
Russula ionochlora	FH 2009 BT01	Germany	MT738288
Russula lakhanpalii	AG 17–1,584	India	MN262088
Russula lakhanpalii	RITF2600	China	MW646983
Russula lavandula	RITF3282	China	OR907087
Russula lavandula	RITF6349	China	OR907084
Russula lilaceofusca	RITF6330	China	OR907102
Russula lilaceofusca	RITF2645	China	OR907093
Russula livescens	H3_10	China	JN129398
Russula livescens	F0177		GU371295
Russula lotus	RITF499	China	MK860699
Russula luofuensis	RITF4708	China	MW646975
Russula maguanensis	XHW4765	China	MH724918
Russula medullata	FH 2011 BT001	Germany	MT738280
Russula medullata	SAV F-1596	Slovakia	MT738281

Russula mustelina	FH12226	Germany	KT934005
Russula mutabilis	DPL10654	USA	KF810137
Russula nigricans	RDL 18-051		OM833135
Russula nigrovirens	HKAS 55222	China	KP171173
Russula nigrovirens	RITF6408	China	OR907095
Russula niveopicta	N.K. Zeng1413 (FHMU958)	China	OP837461
Russula obscuricolor	KD 16-30	IND	MF805816
Russula oleifera	JEIC-0001		MW856423
Russula ombrophila		ESP	KF971694
Russula ornaticeps	MQ21-CMMF005987	Canada	OQ322483
Russula ornaticeps	MQ21-CMMF001660	Canada	OQ322474
Russula pallidirosea	UTC 00274382	USA	NR_153259
Russula pallidula	RITF2613	China	MH027958
Russula parafluvialis	**BJTC FM3332**	**China**	**PX778486**
Russula parafluvialis	**BJTC FM3287**	**China**	**PX778485**
Russula parafluvialis	**BJTC FM1910**	**China**	**PX778484**
Russula parafluvialis	**BJTC FM627 holotype**	**China**	**PX778487**
Russula parvovirescens	SDRM 6280	USA	MK532789
Russula pectinata	2011BT004A	DEU	KF318083
Russula pectinatoides	NYS2303.1	USA	KU640189
Russula perviridis	RITF2912	China	OR907100
Russula perviridis	RITF3131	China	OR907098
Russula phloginea	CNX530524068	China	MK860701
Russula phloginea	CNX530524304	China	MK860700
Russula praetervisa	1997/0812	ITA	UDB019331
Russula prasina	HMAS 281232	China	MH454351
Russula pseudocyanoxantha	*CUH AM177*	*India*	NR_173166
Russula pseudopectinatoides	HMAS251523	CHN	KM269077
Russula pulverulenta	TENN:071739	USA	MG773836
Russula pulverulenta	JLF9697	USA	OQ996326
Russula pulverulenta	12	USA	KJ530747
Russula punctipes	K16051001	CHN	MH168577
Russula putida	IB 1997/0791	ITA	HG798527
Russula quercicola	Russula_sp_ANR_GB55	Pakistan	MZ342771
Russula quercicola	Russula_sp_ANR_GC5	Pakistan	MZ342769
Russula recondita	LUG:19058	Switzerland	NR_147635
Russula recondita	ZT Myc 1704	Switzerland	KF318063
Russula rufobasalis	H17052204	CHN	MH168570
Russula senecis	325-356	JPN	AB509717
Russula shawarensis	LAH36426	Pakistan	MT738293
Russula shawarensis	LAH35453	Pakistan	MT738294
Russula sororia	2010BT234	DEU	KF318052
Russula sp.	Pa1-mOTU086	Japan	LC315895
Russula sp.	6 MAS-2010	Japan	GQ359820

Russula sp.	dc264	Japan	LC538091
Russula subalpinogrisea	KD 18-36	India	MK253443
Russula subalpinogrisea	KD 18-33	India	MK253444
Russula subatropurpurea	K16080818	China	MF433038
Russula subfoetens	5346	ITA	JF908672
Russula subpallidirosea	RITF4083	China	MK860697
Russula subpunicea	RITF3715	China	MN833635
Russula substriata	XHW4785	China	MH724923
Russula substriata	XHW4767	China	MH724922
Russula subterfurcata	RS160510C	Italy	MH285265
Russula swatica	MD14	Pakistan	MK389374
Russula variata	BPL241	USA	KT933959
Russula vesca	RITF5038	China	MW646984
Russula vesca	BPL284	USA	KT933978
Russula vesca	AT2002091	Sweden	DQ422018
Russula vinaceocuticulata	PDD:64246	NZL	GU222258
Russula virescens	HJB9989	Belgium	DQ422014
Russula viridicinnamomea	K15091418	China	MK049972
Russula werneri	IB1997/0786		DQ422021
Russula wulingshanensis	BJTC C403	China	OP133166
Russula wulingshanensis	BTJC L278	China	MW554431
Russula wulingshanensis	BTJC C399	China	OP133165
Russula xanthovirens	H15060611	China	MG786056
Russula xanthovirens	N.K. Zeng3041 (FHMU2002)	China	MT822963
Russulagrisea	2-1129IS75	Europe	AY061679
Russulagrisea	UE2005081601UPS	Sweden	DQ422030
Uncultured Russulaceae	OgaAQ01-aac17d02		HQ330996
