# Supplementary material for: Morphological and Phylogenetic Evidence Reveal Nine New Species of Russula (Russulaceae, Russulales) from Shanxi Province, North China
Source: J Fungi (Basel). 2026 Jan 22;12(1):78. doi: 10.3390/jof12010078 (PMC12843102; doi:10.3390/jof12010078)
Supplement: Supplementary file 1 [file jof-12-00078-s001.zip › Supplement 3.pdf]

Supplement 3. Samples used for multi-locus phylogenetic analysis (*Russula* subgen. *heterophylidia*) and their GenBank accession numbers. Sequences newly generated in this study are in bold. Holotype specimen is marked.

Species	Voucher	Locality	GenBank accession No.	
			ITS	nrLSU
Russula maguanensis	XHW4765	China	MH724918	MH714537
Russula substriata	XHW4766	China	MH724921	MH714540
Russula aeruginea	AT2003017	Sweden	DQ421999	—
Russula aff. crustosa	BB 06.616	Canada	—	KU237461
Russula aff. virescens	BB 09.021	New Caledonia	—	KU237582
Russula albidogrisea	K15091234	China	KY767807	—
Russula albidogrisea	RITF1871	China	MW397095	MW397128
Russula albolutea	RITF2653	China	MT672478	MW397120
Russula albolutea	RITF4460	China	—	MW397121
Russula albolutea	RITF4461	China	—	MW397122
Russula albolutea	RITF4462	China	—	MW397123
Russula amerorecondita	F (PGA17-017)	USA	MN130066	
Russula amerorecondita	SH120828	USA	MN130067	
Russula amoena	SAV F–3147	Slovakia	MT017544	—
Russula atroglauca	HBAU15079	China	MW850413	—
Russula aureoviridis	H16082612	China	KY767809	—
Russula aureoviridis	RITF4709	China	MW646980	MW646992
Russula bella	SFC20170819-05	South Korea	MT017552	—
Russula brevicostata	**BJTC FM1783**	**China**	**PX778479**	**PX778508**
Russula brevicostata	**BJTC FM715 holotype**	**China**	**PX778480**	**PX778509**
Russula bubalina	K15052614	China	MG018742	—
Russula bubalina	RITF1863	China	MW397097	—
Russula cf. crustosa	DSL002	Thailand	MT559557	—
Russula cf. pseudobubalina	HKAS122431	China	ON794290	—
Russula cf. vesca	BB 06.525	Mexico	—	KU237465
Russula crustosa	MHHNU 7960	China	OM760651	—
Russula crustosa	BPL265	United States	KT933966	KT933826
Russula cyanoxantha	FH 12–201	Germany	KR364093	KR364225
Russula cyanoxantha	RITF4682	China	MW646981	MW646993
Russula cyanoxantha	UE29.09.2002–2	France	DQ422033	—
Russula demirimosa	**BJTC FM1006 holotype**	**China**	**PX778481**	**PX778510**
Russula dinghuensis	GDGM45244	China	KU863579	—
Russula dinghuensis	RITF5142	China	MW646982	MW646994
Russula discoidea	N.K. Zeng4895 (FHMU4847)	China	OP837469	OP837459
Russula discoidea	N.K. Zeng4968 (FHMU5535)	China	—	OP837460
Russula dongyaensis	**BJTC FM1718**	**China**	**PX778483**	**PX778512**
Russula dongyaensis	**BJTC FM1753 holotype**	**China**	**PX778482**	**PX778511**
Russula faustiana	15020	USA	JF908705	—
Russula faustiana	FH 2011 BT010	Germany	MT738276	—

Russula fluvialis	KUO (JR8666)	Finland	MN130084	MN130125
Russula fluvialis	KUO (JR8313)	Finland	MN130085	MN130126
Russula galochroa	FH 2010 BT137	Germany	MT738279	MT738255
Russula galochroa	FH 2009 BT02	Germany	MT738278	MT738254
Russula grisea	UE2005.08.16–01	Sweden	DQ422030	—
Russula grisea	FH12234	Germany	KT934006	KT933867
Russula grisea	BB 07.184	Slovakia	—	KU237509
Russula heterophylla	UE20.08.2004–2	Sweden	DQ422006	—
Russula ilicis	563IC52	Europe	AY061682	—
Russula ilicis	MF 00.300	Italy	—	KU237595
Russula ionochlora	BB 07.338	Slovakia	—	KU237508
Russula lakhanpalii	AG 17–1,584	India	MN262088	—
Russula lakhanpalii	RITF2600	China	MW646983	MW646995
Russula langei	BB 07.792	France	—	KU237510
Russula lotus	RITF499	China	MK860699	MW397129
Russula luofuensis	RITF4706	China	MW646973	MW646985
Russula luofuensis	RITF4707	China	MW646974	MW646986
Russula luofuensis	RITF4708	China	MW646975	MW646987
Russula luofuensis	RITF4712	China	MW646976	MW646988
Russula luofuensis	RITF4714	China	MW646977	MW646989
Russula mariae	HCCN19111	South Korea	KF361762	KF361812
Russula mariae	BB 07.038	United States	—	KU237538
Russula medullata	BB 07.252	Slovakia	—	KU237546
Russula mustelina	FH12226	Germany	KT934005	KT933866
Russula mustelina	SA 09.88	Slovakia	—	KU237596
Russula niveopicta	N.K. Zeng1413 (FHMU958)	China	OP837461	OP837453
Russula niveopicta	N.K. Zeng1395 (FHMU941)	China	OP837462	OP837454
Russula niveopicta	N.K. Zeng2252 (FHMU1497)	China	OP837463	OP837455
Russula niveopicta	N.K. Zeng1408 (FHMU953)	China	OP837464	OP837456
Russula ombrophila		ESP	KF971694	
Russula orientipurpurea	SFC20170819-08	South Korea	MT017550	—
Russula orientipurpurea	SFC20170725-37	South Korea	MT017548	—
Russula pallidula	RITF2613	China	MH027958	MH027960
Russula pallidula	RITF3331	China	MH027959	MH027961
Russula parafluvialis	**BJTC FM3332**	**China**	**PX778486**	—
Russula parafluvialis	**BJTC FM3287**	**China**	**PX778485**	**PX778514**
Russula parafluvialis	**BJTC FM1910**	**China**	**PX778484**	**PX778513**
Russula parafluvialis	**BJTC FM627 holotype**	**China**	**PX778487**	—
Russula parvovirescens	SDRM 6280	United States	MK532789	—
Russula phloginea	CNX530524068	China	MK860701	MK860704
Russula phloginea	CNX530524304	China	MK860700	MK860703
Russula prasina	HMAS 281232	China	MH454351	—
Russula prasina	HMAS 279806	China	MH454353	—
Russula prasina	HMAS 279805	China	MH454352	—

Russula pseudobubalina	GDGM70632	China	MF433036	—
Russula quercicola	Russula_sp_ANR_GB55	Pakistan	MZ342771	MZ342774
Russula quercicola	Russula_sp_ANR_GC5	Pakistan	MZ342769	
Russula recondita	LUG:19058	Switzerland	NR_147635	
Russula recondita	ZT Myc 1704	Switzerland	KF318063	
Russula shawarensis	LAH36426	Pakistan	MT738293	MT738268
Russula shawarensis	LAH35453	Pakistan	MT738294	MT738269
Russula sp.	Pj3-mOTU063	Japan	LC260471	—
Russula sp.	Pa1-mOTU086	Japan	LC315895	—
Russula sp.	TY613	Japan	LC367995	—
Russula sp.	Pj3-mOTU065	Japan	LC260473	—
Russula sp.	HMAS:279584	China	MG719936	—
Russula sp.	HMAS 276811	China	LT602970	LT602947
Russula sp.	6 MAS-2010	Japan	GQ359820	—
Russula sp.	B4-1	Japan	LC553324	—
Russula sp.	dc264	Japan	LC538091	—
Russula sp.	TJS2020-03	China	OM281259	OM281030
Russula sp.	TYY-73	China	OK584446	—
Russula sp.	1734	China	AB769908	—
Russula sp.	HMAS:271715	China	KX441239	KX441486
Russula subatropurpurea	N.K. Zeng4898 (FHMU4841)	China	OP837465	—
Russula subatropurpurea	N.K. Zeng4910 (FHMU4854)	China	OP837467	OP837457
Russula subatropurpurea	N.K. Zeng5034 (FHMU4812)	China	OP837468	OP837458
Russula subatropurpurea	N.K. Zeng4764 (FHMU5454)	China	OP837466	—
Russula subatropurpurea	K16080818	China	MF433038	—
Russula subatropurpurea	K16080816	China	MF433037	—
Russula subatropurpurea	K17071401	China	MH422579	—
Russula subbubalina	RITF4710	China	MW646978	MW646990
Russula subbubalina	RITF4715	China	MW646979	MW646991
Russula subfoetens	5346	ITA	JF908672	
Russula subpallidirosea	RITF4083	China	MK860697	MK860702
Russula subpunicea	RITF3715	China	MN833635	MW397124
Russula subpunicea	RITF2648	China	MN833638	MW397125
Russula subpunicea	RITF1435	China	MN833637	MW397126
Russula subpunicea	RITF2615	China	MN833636	MW397127
Russula subterfurcata	RS160510C	Italy	MH285265	
Russula variata	BPL241	United States	KT933959	KT933818
Russula vesca	RITF5038	China	MW646984	—
Russula vesca	BPL284	United States	KT933978	KT933839
Russula vesca	AT2002091	Sweden	DQ422018	—
Russula violeipes	BB 07.273	Slovakia	—	KU237534
Russula violeipes	SFC20121010-06	South Korea	KF361808	KF361858
Russula virescens	HJB9989	Belgium	DQ422014	—
Russula viridicinnamomea	K15091418	China	MK049972	—

Russula viridicinnamomea	RITF3324	China	MW397098	MW397130
Russula viridirubrolimbata	HBAU 15011	China	MT337526	—
Russula werneri	IB1997/0786	Europe	DQ422021	—
Russula xanthovirens	GDGM 71145	China	MG786056	—
Russula xanthovirens	N.K. Zeng3025 (FHMU1986)	China	—	OP837452
Russula xanthovirens	N.K. Zeng3041 (FHMU2002)	China	MT822963	MT829148
Russula xanthovirens	B17091630	China	MG786055	—
