# Supplementary material for: Morphological and Phylogenetic Evidence Reveal Nine New Species of Russula (Russulaceae, Russulales) from Shanxi Province, North China
Source: J Fungi (Basel). 2026 Jan 22;12(1):78. doi: 10.3390/jof12010078 (PMC12843102; doi:10.3390/jof12010078)
Supplement: Supplementary file 1 [file jof-12-00078-s001.zip › Supplement 4.pdf]

**Supplement 4.** Samples used for ITS phylogenetic analysis (*Russula* subgen. *russula*) and their GenBank accession numbers. Sequences newly generated in this study are in bold. Holotype specimen is marked.

| Species | Voucher | Locality | GenBank accession No. ITS |
|---|---|---|---|
| *Multifurca zonaria* | DED7442 | | DQ421990 |
| *Multifurca ochricompacta* | BB02107 PC | | DQ421984 |
| *Russula aff. pascua* | CRN 138 | USA | MT583275 |
| *Russula aquosa* | 312RUF25 | Europe | AY061657 |
| *Russula aurantioflammans* | r3245 | Slovakia | KU928167 |
| *Russula aurantioflammans* | MQ21-HL0994-QFB32789 | Canada | OQ322258 |
| *Russula aurantioflava* | LAH35409 (GJ1655) | Pakistan | MN130071 |
| *Russula aurantioflava* | LAH35408 (GJ1640) | Pakistan | MN130070 |
| *Russula aurata* | 21120IS77 | Europe | AY061659 |
| *Russula begonia* | HBAU15565 | China | MZ573247 |
| *Russula begonia* | HBAU15564 | China | MZ573252 |
| *Russula begonia* | CFSZ2192 | China | MZ573264 |
| *Russula begonia* | CFSZ20023 | China | MZ573267 |
| *Russula candidissima* | JMV201109066a BCN | Spain | MK105636 |
| *Russula cessans* | | Canada | LC192757 |
| *Russula clariana* | 492RUS26 | Europe | AY061664 |
| *Russula clavatohyphata* | AG 16 1223 | India | MG934210 |
| *Russula clavatohyphata* | AG 15 756 | India | MG934209 |
| *Russula clavipes* | SAVF1327 | Slovakia | KU205292 |
| *Russula cornicolor* | FH18154 | Panama | MW058808 |
| *Russula cornicolor* | AC494 | Panama | MW084356 |
| *Russula coronaspora* | GDGM79711 | China | MN275689 |
| *Russula coronaspora* | GDGM79712 | China | MN275690 |
| *Russula cremeirosea* | BPL289 | USA | KT933983 |
| *Russula cremeirosea* | JMP0061 | USA | EU819424 |
| *Russula cuprea* | FH12250 | Germany | KT934010 |
| *Russula cuprea* | 2-1127IS77 | USA | AY061667 |
| *Russula curtipes* | 1123IS77 | Europe | AY061668 |
| *Russula curtipes* | FH12206 | Germany | KT933995 |
| *Russula cynorhodon* | FH18117 | Panama | MW058812 |
| *Russula cynorhodon* | FH18118 | Panama | MW058809 |
| *Russula dryadicola* | 16243 | USA | JF908710 |
| *Russula emetica* | UE05.10.2003-11 | Sweden | DQ421997 |
| *Russula emetica* | lw81 (TUB) | Germany | AF418619 |
| *Russula emetica* | 517IS76 | USA | AY061673 |
| *Russula exalbicans* | 21117IS76 | Europe | AY061674 |
| *Russula faginea* | SAV:F-1337 | France | KU205289 |
| *Russula faginea* | SAV:F-997 | Slovakia | KU205286 |

| | | | |
|---|---|---|---|
| *Russula fanjing* | HGASMF0110047 | China | MT928349 |
| *Russula fanjing* | HBAU15040 | China | MT928356 |
| *Russula flavida* | PC BB2004250 | USA | EU598171 |
| *Russula font-queri* | fruit body93 | China | MN704820 |
| *Russula font-queri* | FH12223 | Germany | KT934003 |
| *Russula fragilis* | MQ17160-QFB29668 | Canada | MN992642 |
| *Russula fragilis* | QHU20085 | China/Qinghai | OM970894 |
| *Russula globispora* | SAV:HK12021 | Slovakia | KU886595 |
| *Russula globispora* | GENT:FH-2007-BT121 | Germany | KU886594 |
| *Russula gracillima* | 483IS76 | USA | AY061678 |
| *Russula gracillima* | FH 12-264 | Germany | KR364094 |
| *Russula graveolens* | SAV:F-1339 | Belgium | KU205301 |
| *Russula graveolens* | SAV:F-1343 | Slovakia | KU205298 |
| *Russula heilongjiangensis* | HMAS255142 | China | MG719932 |
| *Russula heilongjiangensis* | HMAS279587 | China | MG719933 |
| *Russula helodes* | 497RUF26 | Europe | AY061680 |
| *Russula hypofragilis* | UWBMWTUF038403 | USA | KX813553 |
| *Russula integriformis* | 561IS78 | Europe | AY061684 |
| *Russula intermedia* | SAVF3093 | Norway | KU928147 |
| *Russula jilinensis* | HMAS194253 | China | GU966632 |
| *Russula khinganensis* | HMAS279576 | China | MG719929 |
| *Russula khinganensis* | HMAS278895 | China | MG719928 |
| *Russula laccata* | CLC_1378 | USA | MT583295 |
| *Russula laccata* | CLC 3617 | USA | MT583209 |
| *Russula laeta* | PRM 945739 | Czech Republic | MG679812 |
| *Russula lepidicolor* | 493RUF26 | Europe | AY061687 |
| *Russula leucomarginata* | RITF3133 | China | MW301626 |
| *Russula leucomarginata* | RITF3123 | China | MW301627 |
| ***Russula liuboanum*** | **BJTC FM2973** | **China** | **PX778491** |
| ***Russula liuboanum*** | **BJTC FM2255** | **China** | **PX778489** |
| ***Russula liuboanum*** | **BJTC FM3446** | **China** | **PX778495** |
| ***Russula liuboanum*** | **BJTC FM2969** | **China** | **PX778490** |
| ***Russula liuboanum*** | **BJTC FM982** | **China** | **PX778488** |
| ***Russula liuboanum*** | **BJTC FM3032** | **China** | **PX778492** |
| ***Russula liuboanum*** | **BJTC FM3323** | **China** | **PX778493** |
| ***Russula liuboanum*** | **BJTC FM3439 holotype** | **China** | **PX778494** |
| *Russula lutea* | HMJAU 38003 | China | KY681442 |
| *Russula lutea* | OSA:MY-7810 | Japan | LC192788 |
| *Russula mattiroloana* | GK3901, JMV800638 (BCN) | Greece | MK105652 |
| *Russula mattiroloana* | F-2017-1 (KRA), JMV800670 (BCN) | Poland | MK105655 |
| *Russula mediterraneensis* | MG630 29085 MCVE | Italy | MK105661 |
| *Russula mediterraneensis* | MG636 29086 MCVE | Italy | MK105662 |
| *Russula meridionalis* | JC180617NR | Spain | MK105667 |

| | | | |
|---|---|---|---|
| *Russula messapica* | 562IC52 | Europe | AY061692 |
| *Russula minor* | GDGM79686 | China | MN275666 |
| *Russula minor* | GDGM79687 | China | MN275665 |
| *Russula miyunensis* | BJTC ZH1357 | China | OP133218 |
| *Russula miyunensis* | BJTC Z1355 | China | OP133219 |
| *Russula nauseosa* | FH12173 | Germany | KT933985 |
| *Russula nauseosa* | CM10 | China | MN240848 |
| *Russula nauseosa* | CM11 | China | MN240849 |
| *Russula nitida* | KR:0004221 | Germany | KU205349 |
| *Russula nitida* | R91 | Czech Republic | MG679818 |
| *Russula nuoljae* | SAVF3092 | Norway | KU205350 |
| *Russula obscurozelleri* | UWBMWTUF039112 | USA | KX813502 |
| *Russula obscurozelleri* | UWBMWTUF038663 | USA | KX813466 |
| *Russula odorata* | 526 07186 | Slovakia | JN944010 |
| *Russula odorata* | 1113IS75 | Europe | AY061698 |
| *Russula olivacea* | hue85 (TUB) | Germany | AF418634 |
| *Russula oreomunneae* | FH18151 | Panama | MW058804 |
| *Russula oreomunneae* | AC190 | Panama | KM594825 |
| *Russula paragraveolens* | ZRL20160546 | China | OQ871504 |
| *Russula paragraveolens* | ZRL20162647 | China | OQ871505 |
| *Russula paraxerampelina* | CFSZ 21050(htp) | China | OR520399 |
| *Russula paraxerampelina* | CFSZ 20782 | China | OR520398 |
| *Russula pelargonia* | JMT-20093016 | France | PV742609 |
| *Russula pelargonia* | JMT-19102806 | France | PV742608 |
| *Russula persicina* | UE 21.09.2003-01 | Sweden | UDB002502 |
| *Russula persicina* | TU F101826 | Estonia | UDB015984 |
| *Russula plana* | BJTC ZH1398 | China | OP133220 |
| *Russula plana* | BJTC T2101 | China | OP263722 |
| *Russula pseudograveolens* | ZRL20211703 | China | OQ871496 |
| *Russula pseudograveolens* | ZRL20211685 | China | OQ871497 |
| *Russula pseudosinoparva* | BJTC C381 | China | OR056321 |
| *Russula pseudosinoparva* | BJTC L131 | China | OR056320 |
| *Russula pseudotsugarum* | UWBM:WTU-F-038562 | USA | KX813578 |
| *Russula puellaris* | 21099IS72 | Europe | AY061709 |
| *Russula puellaris* | nl1372 | Germany | AF418628 |
| *Russula puellula* | 21130IS76 | Europe | AY061710 |
| *Russula pusilla* | BPL267 | USA | KT933968 |
| ***Russula puxianensis*** | **BJTC FM3038** | **China** | **PX778501** |
| ***Russula puxianensis*** | **BJTC FM1899** | **China** | **PX778498** |
| ***Russula puxianensis*** | **BJTC FM2392** | **China** | **PX778499** |
| ***Russula puxianensis*** | **BJTC FM709** | **China** | **PX778496** |
| ***Russula puxianensis*** | **BJTC FM3033** | **China** | **PX778500** |
| ***Russula puxianensis*** | **BJTC FM1868 holotype** | **China** | **PX778497** |
| *Russula roseola* | RITF3428 | China | MW301619 |

| | | | |
|---|---|---|---|
| ***Russula rubrolivacea*** | **BJTC FM1821_1** | **China** | **PX778502** |
| ***Russula rubrolivacea*** | **BJTC FM1821 holotype** | **China** | **PX778503** |
| *Russula ryukokuensis* | TNSF70424 | Japan | MH037291 |
| *Russula ryukokuensis* | TNSF70425 | Japan | MH037292 |
| *Russula saliceticola* | CRN 134 | USA | MT583225 |
| *Russula saliceticola* | CLC_2370 | USA | MT583282 |
| *Russula sanguinea* | FH12240 | Germany | KT934008 |
| *Russula sichuanensis* | HKAS 53885 | China | JX391968 |
| *Russula sichuanensis* | HKAS53792 | China | JX391969 |
| ***Russula sinocurtipes*** | **BJTC FM3291** | **China** | **PX778505** |
| ***Russula sinocurtipes*** | **BJTC FM2493 holotype** | **China** | **PX778504** |
| *Russula sinoparva* | BJTC C540 | China | MW554236 |
| *Russula sinoparva* | BJTC Z441 | China | OP133221 |
| *Russula sinorobusta* | BJTC Z050 | China | OP133222 |
| *Russula sinorobusta* | BJTC Z052 | China | OP133223 |
| *Russula sinorobusta* | BJTC Z662 | China | OP133224 |
| *Russula solaris* | 559 07282 | Slovakia | JN944007 |
| *Russula solaris* | hue219 | Germany | AF418627 |
| *Russula subrubens* | CLC 1218 | USA | MT583232 |
| *Russula subrubens* | CLC 1219 | USA | MT583233 |
| *Russula subsanguinaria* | RITF2236 | China | MW301621 |
| *Russula subsanguinaria* | RITF2208 | China | MW301622 |
| *Russula subversatilis* | BJTC C653 | China | MW554190 |
| *Russula subversatilis* | BJTC T2001 | China | OP263723 |
| *Russula tengii* | HMAS 262728 | China | NR_169926 |
| *Russula thindii* | RITF2722 | China | KU290399 |
| *Russula torulosa* | IZS127B | Italy | MZ005531 |
| *Russula torulosa* | IZS127A | Italy | MZ005530 |
| *Russula uttarakhandia* | AG 161190 | India | MF684758 |
| *Russula uttarakhandia* | CAL 1537 | India | KY873997 |
| *Russula velenovskyi* | 526IS77 | Europe | AY061721 |
| *Russula versicolor* | 589 07288 | Slovakia | JN944009 |
| *Russula versicolor* | 320RUS25 | Europe | AY061722 |
| *Russula veternosa* | SAV F2588 | Slovakia | KY582699 |
| *Russula veternosa* | SAV F1491 | Slovakia | KY582685 |
| *Russula vinaceodora* | 46374 AH | Spain | MK105695 |
| *Russula vinosobrunneola* | HMAS 281138 | China | NR_160502 |
| *Russula vinosobrunneola* | HMAS278960 | China | MG719926 |
| *Russula vinosobrunneola* | HMAS 278885 | China | MG719925 |
| *Russula violacea* | 322IS55 | Europe | AY061725 |
| *Russula wielangtae* | HO 593334 | Australia | MN130116 |
| *Russula xerampelina* | SAV F-2260 | Slovakia | OR198784 |
| *Russula xerampelina* | SAV F-2258 | Slovakia | OR198786 |
| *Russula xerophila* | OSC82218 | USA | NR_119557 |

| | | | |
|---|---|---|---|
| *Russula yadongensis* | HMAS287387 | China | OQ871499 |
| *Russula yadongensis* | HMAS287386 | China | OQ871498 |
| *Russula yadongensis* | HMAS287388 | China | OQ871500 |
| *Russula yanshanensis* | BJTC Z1305 | China | OP133228 |
| *Russula yanshanensis* | BJTC Z1390 | China | OP133229 |
| *Russula zelleri* | NYGB-761009 | USA | KX812833 |
| *Russula zelleri* | D.Bojantchev 129 | USA | JF834326 |
| *Russula zephyrovelutipes* | FH18116 | Panama | MW058806 |
| *Russula zephyrovelutipes* | FH18051 | Panama | MW058807 |
| *Russula zvarae* | PCBartBuyck08639 | France | JN944004 |
| *Russula zvarae* | PCBartBuyck08668 | Italy | JN944002 |
