# Supplementary material for: Morphological and Phylogenetic Evidence Reveal Nine New Species of Russula (Russulaceae, Russulales) from Shanxi Province, North China
Source: J Fungi (Basel). 2026 Jan 22;12(1):78. doi: 10.3390/jof12010078 (PMC12843102; doi:10.3390/jof12010078)
Supplement: Supplementary file 1 [file jof-12-00078-s001.zip › Supplement 5.pdf]

Supplement 5. Samples used for multi-locus phylogenetic analysis (*Russula* subgen. *russula*) and their GenBank accession numbers. Sequences newly generated in this study are in bold. Holotype specimen is marked.

Species	Voucher	Locality	GenBank accession No.		
			LSU	*rpb2*	*tef-1*
Multifurca aurantiophylla	644/BB 09.119	New Caledonia	KU237581	KU237867	KU238008
Multifurca ochricompacta	580/BB 07.010	USA	KU237565	KU237851	KU237994
Russula abbottabadensis	LAH 310071	Pakistan	MN518356	MG386737	MZ364137
Russula abbottabadensis	FH 00304558	Pakistan	MN518355	MG386738	MZ364138
Russula acrifolia	543/BB 08.662	Italy	KU237535	KU237821	KU237965
Russula adusta	223/BB 06.562	Canada	KU237476	KU237762	KU237907
Russula amethystina	529/BB 07.314	Slovakia	KU237521	KU237807	KU237951
Russula archaeosuberis	1118/BB 12.085	Italy	KU237593	KU237878	KU238019
Russula ayubiana	LAH 35438	Pakistan	MZ358816	MZ364131	MZ364139
Russula ayubiana	LAH 35439	Pakistan	MZ358817	MZ364132	MZ364140
Russula azurea	537/BB 08.668	Italy	KU237529	KU237815	KU237959
Russula betularum	BPL269	USA	KT933829	KT933900	-
Russula brevipes	226/BB 06.508	Mexico	KU237479	KU237765	-
Russula burlinghamiae	548/BB 05.108	USA	KU237540	KU237826	KU237970
Russula carpini	551/BB 07.262	Slovakia	KU237543	KU237829	KU237973
Russula chloroides	572/BB 07.209	Slovakia	KU237559	KU237845	KU237990
Russula compacta	228/BB 06.295	USA	KU237480	KU237766	-
Russula corallina-	229/BB 06.324	USA	KU237481	KU237767	KU237910
Russula crustosa	BPL265	USA	KT933826	KT933898	-
Russula cuprea	565/BB 07.233	Slovakia	KU237555	KU237841	KU237984
Russula curtipes	1123IS77	Europe			
Russula curtipes	FH12206	Germany	KT933856	KT933927	
Russula decolorans	549/BB 07.322	Slovakia	KU237541	KU237827	KU237971
Russula emetica	635/JMT39-08092228	France	KU237578	KU237864	-
Russula exalbicans	584/BB 07.786	France	KU237568	KU237854	KU237996
Russula farinipes	576/BB 08.632	Italy	KU237561	KU237847	KU237992
Russula fattoensis	Buyck 02.227	USA	MN315514	MN326797	MN326800
Russula font-queri	fruit body93	China	MN710560		
Russula font-queri	FH12223	Germany	KT933864	KT933935	
Russula fragilis	443/BB 07.791	France	KU237506	KU237792	-
Russula glutinosa	Roody WRWV 04.1154	USA	MN315511	MN326798	MN326799
Russula gracillima	441/BB 07.785	France	KU237504	KU237790	KU237934
Russula griseobrunnea	JAC11227	New Zealand	MW683630		
Russula heilongjiangensis	HMAS255142	China	MH021611		
Russula heilongjiangensis	HMAS279587	China	MH021614		
Russula heilongjiangensis	HMAS255142	China	MH02161		
Russula heilongjiangensis	HMAS279587	China	MH021614		
Russula herrerae	239/BB 06.532	Mexico	KU237486	KU237772	KU237915
Russula integra	518/BB 07.198	Slovakia	KU237513	KU237799	KU237943

Russula integriformis	561IS78	Europe			
Russula laeta	519/BB 07.267	Slovakia	KU237514	KU237800	KU237944
Russula laeta	PRM 945739	Czech Republic			
Russula laricina	575/BB 08.681	Italy	KU237560	KU237846	KU237991
Russula leucomarginata	RITF3133	China	MW309327	MW310568	-
Russula leucomarginata	RITF3123	China	MW309328	MW310569	-
Russula lilacea	435/BB 07.213	Slovakia	KU237498	KU237784	KU237928
Russula liuboanum	**BJTC FM2969**	**China**	**PX778515**	**PX789037**	**PX829013**
Russula liuboanum	**BJTC FM3446**	**China**	**PX778516**	**PX789038**	**PX829014**
Russula liuboanum	**BJTC FM3439 *holotype***	**China**	**This study**	**PX789039**	**PX829015**
Russula mansehraensis	HUP SUR 180	Pakistan	MG944280	MG944255	-
Russula mansehraensis	HUP SUR 803	Pakistan	-	MG944256	-
Russula minutula	539/BB 08.636	Italy	KU237531	KU237817	KU237961
Russula miyunensis	BJTC Z1355	China	OP133232	OP156827	-
Russula miyunensis	BJTC Z1357	China	-	OP156826	OP156837
Russula mustelina	1176/SA 09.88	Slovakia	KU237596	KU237881	KU238022
Russula nauseosa	588/BB 07.285	Italy	KU237572	KU237858	KU238000
Russula nigricans	429/BB 07.342	Slovakia	KU237495	KU237781	KU237924
Russula nitida	KR:0004221	Germany			
Russula nitida	PRM 922543	Czech Republic			
Russula nothofagineae	723/BB 09.044	New Caledonia	KU237583	-	KU238010
Russula nothofagineae	726/BB 09.069	New Caledonia	KU237585	KU237870	KU238012
Russula odorata	526/BB 07.186	Slovakia	KU237518	KU237804	KU237948
Russula olivacea	hue85 (TUB)	Germany	AF325314		
Russula olivascens	530/BB 08.663	Italia	KU237522	KU237808	KU237952
Russula olivobrunnea	JV28388	Finland	-	-	-
Russula plana	BJTC Z1398	China	OP133233	OP156828	OP156838
Russula plana	BJTC T2101	China	OP265903	OP267556	OP267558
Russula pseudoaurantiophylla	740/BB 09.219	New Caledonia	KU237591	KU237876	KU238017
Russula puellaris	523/BB 07.311	Slovakia	KU237515	KU237801	KU237945
Russula purpureoverrucosa	GDGM32902	China	MG214699	MT085652	MT085623
Russula pusilla	BPL267	USA	KT933828		
Russula puxianensis	**BJTC FM1899**	**China**	**PX778518**	-	**PX829012**
Russula puxianensis	**BJTC FM2932**	**China**	**PX778519**	**PX789040**	-
Russula puxianensis	**BJTC FM1868 *holotypoe***	**China**	**PX778517**	-	-
Russula raoultii	561/BB 08.674	Italy	KU237551	KU237837	KU237980
Russula rosea	430/BB 07.780	France	KU237496	KU237782	KU237925
Russula roseola	RITF3418	China	MW309319	MW310560	-
Russula roseola	RITF3428	China	MW309320	MW310561	-
Russula rubrolivacea	**BJTC FM1821_1**	**China**	**PX778520**	**PX789042**	**PX829017**
Russula rubrolivacea	**BJTC FM1821 *holotype***	**China**	**PX778521**	**PX789043**	**PX829016**

Russula saliceticola	CRN 134	USA		MT500716	
Russula saliceticola	CLC_2370	USA		MT500719	
Russula sichuanensis	ZRL20162017	China	MG786572	-	MG812160
Russula sinocurtipes	**BJTC FM3291**	**China**	**PX778523**	**PX789044**	-
Russula sinocurtipes	**BJTC FM2493 *holotype***	**China**	**PX778522**	**PX789043**	-
Russula sinoparva	BJTC C540	China	OP133234	OP156829	OP156839
Russula sinoparva	BJTC Z441	China	OP133235	-	OP156840
Russula sinorobusta	BJTC Z050	China	OP133236	OP156830	OP156841
Russula sinorobusta	BJTC Z052	China	-	-	OP156842
Russula sinorobusta	BJTC Z662	China	OP133237	OP156831	OP156843
Russula solaris	559/BB 07.282	Slovakia	KU237549	KU237835	KU237978
Russula sp.	735/BB 09.172	New Caledonia	KU237588	KU237873	KU238015
Russula subsanguinaria	RITF2236	China	MW309322	MW310563	-
Russula subsanguinaria	RITF2208	China	MW309323	MW310564	-
Russula subtilis	536/BB 05.107	USA	KU237528	KU237814	KU237958
Russula subversatilis	BJTC C653	China	OP133238	OP156832	OP156844
Russula subversatilis	BJTC T2001	China	OP265904	OP267557	OP267559
Russula turci	528/BB 07.328	Slovakia	KU237520	KU237806	KU237950
Russula velenovskyi	526IS77	Europe			
Russula versicolor	589/BB 07.288	Slovakia	KU237573	KU237859	KU238001
Russula veternosa	SAV F2588	Slovakia		KY616693	
Russula veternosa	SAV F1491	Slovakia		KY616681	
Russula vinosobrunneola	HMAS 281138	China	MG786569	-	MG812157
Russula vinosobrunneola	HMAS 278885	China	MG786570	-	MG812158
Russula yanshanensis	BJTC Z1448	China	OP133240	OP156833	-
Russula yanshanensis	BJTC C561	China	OP133239	-	OP156845
Russula yanshanensis	BJTC Z421	China	OP133241	-	OP156846
Russula yanshanensis	BJTC Z1385	China	OP133242	-	OP156847
Russula yanshanensis	BJTC Z1305	China	OP133243	OP156834	OP156848
Russula yanshanensis	BJTC Z1390	China	OP133244	OP156835	OP156849
Russula yanshanensis	BJTC L349	China	OP133245	OP156836	OP156850
Russula zvarae	538/BB 08.639	Italy	KU237530	KU237816	KU237960
